# Design of nucleic acid strands with long low-barrier folding pathways

**DOI:** 10.1007/s11047-016-9587-9

**Published:** 2017-01-03

**Authors:** Anne Condon, Bonnie Kirkpatrick, Ján Maňuch

**Affiliations:** 1 Department of Computer Science, University of British Columbia, Vancouver, Canada; 2 Intrepid Net Computing, Dillon, MT USA

**Keywords:** Nucleic acid strands, Low-barrier pathways, Sequence design, Folding pathways

## Abstract

A major goal of natural computing is to design biomolecules, such as nucleic acid sequences, that can be used to perform computations. We design sequences of nucleic acids that are “guaranteed” to have long folding pathways relative to their length. This particular sequences with high probability follow low-barrier folding pathways that visit a large number of distinct structures. Long folding pathways are interesting, because they demonstrate that natural computing can potentially support long and complex computations. Formally, we provide the first scalable designs of molecules whose low-barrier folding pathways, with respect to a simple, stacked pair energy model, grow superlinearly with the molecule length, but for which all significantly shorter alternative folding pathways have an energy barrier that is $2 - \epsilon$ times that of the low-barrier pathway for any $\epsilon > 0$ and a sufficiently long sequence.

## Introduction

Novel means for performing computations or designing nanostructures at the molecular level have been successfully developed, that exploit base pairing interactions of nucleic acids. Prominent examples include DNA strand displacement systems (DSDs) (Seelig et al. 2006) and RNA origami systems (Geary and Andersen 2014). Our work here is motivated by the goal of computing with a single RNA sequence as the nucleic acids of the sequence interact with each other.

RNA sequences form folded structures in which pairs of nucleic acids biochemically bond to each other. These bonds change the physical energy of the sequence, and a given sequence prefers to assume low-energy folded structures. Folding is a dynamic process, constrained by kinetics, during which an RNA sequence will move through a sequence of structures with each differing from the previous one by the addition or removal of a single base pair. The process may reach a low-energy structure from a high-energy structure or simply maintain low energy (a process referred to as the natural “breathing” of the molecule). Folding pathways will tend to meander along low-energy “valleys” in the landscape of secondary structures, rather than scaling high-energy “barriers”, even if the low-barrier valleys are longer.

Here, we focus on design of an RNA sequence that traverses a low-energy pathway as the molecule breathes. We imagine molecules suspended in a solution, where each molecule interacts only with itself. Our goal is to design nucleic acid strands that, because of kinetic folding constraints, are fated to follow *inordinately long* low-barrier pathways, relative to the strand length, from some initial to target structure. We seek a scalable design that, for any $n$, yields a strand of length $\varTheta (n)$ such that all low-barrier pathways from initial to target visit a number of distinct structures that grows superlinearly with $n$, while any shorter pathway has a significantly higher barrier and is unfavourable kinetically.

### Motivation and related work

The motivation for our goal stems in part from the strengths and weaknesses of multi-stranded nucleic acid systems, such as DNA strand displacement systems (DSDs), as a means of molecular programming and design. Toehold-mediated DNA strand displacement systems support circuit and artificial neural network computations via folding pathways (Qian and Winfree 2011; Qian et al. 2011; Seelig et al. 2006), and can in principle support general Turing machine computations (Qian et al. 2011). Earlier designs of multi-state DNA machines also relied on multi-stranded folding pathways involving the formation and breakage of hairpins (Hagiya et al. 2006; Uejima and Hagiya 2004). Moreover, multi-stranded DNA folding pathways are the means for realizing molecular tweezers (Yurke et al. 2000), autonomous locomotors (Yin et al. 2008), and many other nano-scale mechanical devices (Simmel and Dittmer 2005). In these examples, correct steps in a computation or execution of a device correspond to low-barrier pathways of multi-stranded pseudoknot-free structures; incorrect steps are unfavourable because of high energy barriers. However, all of these computational or mechanical processes use a number of strands that is proportional to the number of steps of the process. Consider, for example, computations involving strand displacement, in which so-called signal strands serve as the memory of the computation. Each signal strand is a reactant in only one strand displacement step, becoming part of a waste complex that is one of the products of the step. Thus, DNA strand displacement processes use DNA strands as a sort of write-once, read-once memory. If such a process occurs in a closed volume, that volume must be at least proportional to the number of steps of the process in order to accommodate all of the needed strands. This is very different from typical silicon-based computations, where memory can be re-used.

DSDs can in principle simulate volume-efficient computations, i.e., computations where the total length of strands involved is polynomial in the input size, via multi-stranded folding pathways that have length exponential in the number of strands involved (Thachuk and Condon 2012). However, such volume-efficient DSD computations would be difficult to carry out experimentally, in part because single copies of some participating strands are needed. To avoid this difficulty and other practical limitations of multi-stranded DSDs, it would be interesting to find a way to compute in a volume-efficient way within a single strand. A computation would correspond to a folding pathway of the strand from some input structure to a solution structure; the longer the pathway, the longer the computation.

Apart from this computational motivation, developing principles for design of nucleic acids whose structure or kinetically-preferred folding pathways have unusual properties can contribute to fundamental scientific understanding of the diversity of folding pathways that are possible with the basic building blocks of nature and, ultimately, applications of this diversity. There has been much interest in inverse RNA secondary structure prediction, that is, computational design of RNA sequences that fold into given (typically pseudoknot free) secondary structures (Andronescu et al. 2004; Busch and Backofen 2006; Dirks et al. 2004; Haleš et al. 2015; Jaeger et al. 2001; Leea et al. 2014; Schuster et al. 1994; Zhou et al. 2013). Leea et al. (2014) have developed eteRNA, a crowd-sourcing approach to RNA secondary structure design and inference of design rules. Mathieson and Condon (2015) provide designs of RNA sequences with folding pathway whose minimum barrier pathways from an initial to target structure are necessarily indirect, that is, involve base pairs that are neither in the initial or target structure, and in addition may contain “repeats” where a base pair is removed and later added back in again. The folding pathways introduced in this paper contain structures with both indirect repeated base pairs, with the repetitions occurring many times, in contrast to just one repeat in the designs of Mathieson and Condon.

Yet other related work pertains to the design of bistable or multistable DNA or RNA molecules, inspired by biological molecular switches. Molecular riboswitches are bistable RNA molecules in nature that are capable of changing structure, and thus function, in changing environments; there is evidence that molecular switches facilitate processes such as viroid replication (Gultyaev et al. 1998) and gene expression (Babitzke and Yanofsky 1993). Goals in the field of synthetic biology and its applications have motivated rational computational design of synthetic riboswitches—subsequences of mRNA’s that regulate gene expression via structural changes—sometimes guided by properties of the RNA’s folding pathways (Beisel and Smolke 2009; Isaacs et al. 2006). Soukup and Breaker (1999) designed an RNA switch that changes its structure in the presence of certain ligands. Schultes and Bartel (2000) designed an RNA sequence whose bistable structures are motifs of two functionally different ribozymes, even though the two structures have no base pairs in common. There has also been work on design (or redesign) of protein folding pathways (Kuhlman et al. 2002; Nauli et al. 2001), motivated both by improving fundamental understanding of protein folding pathway processes and also by the goal of designing proteins that fold into biologically-relevant structures with faster folding rates than wild-type protein sequences.

Flamm et al. (2000) show how the design of multi-stable nucleic acid sequences can be cast as an optimization problem with constraints, and have developed computational methods to design sequences that satisfy the constraints. Their methods can be used, for example, to design a sequence with two prescribed low-energy structures and a high energy barrier between these structures. The design goal that we consider here, namely to design sequences with long folding pathways, is quite different than the design goals for multi-stable sequences or switches, but our design incorporates both of these elements in more general ways than previous work. Specifically, our sequences have multiple stable (i.e., minimum free energy) structures—in fact, the number of such structures grows as a function of the overall sequence length, unlike designs proposed by Schultes and Bartel and by Flamm et al. Our design also incorporates a switch, whose purpose is to provide relatively low-barrier pathways between numerous minimum free energy structures of the rest of our sequence. In contrast, the Soukup-Breaker design facilitates a switch between just two configurations. Of course, while our design incorporates switching and multi-stability in more general ways than previous work, we can only establish its efficacy on paper, with respect to a simple energy model. Moreover, we provide just one design, whereas Flamm et al. provide a design method which can produce many designs with different constraints.

In the field of nucleic acid nanotechnology, Geary et al. (2014), Geary and Andersen (2014) recently showed how to create RNA origami structures using single strands. In contrast with earlier DNA origami folding, where short staple strands guide the folding pathway of a much longer DNA strand, Geary et al. use co-transcriptional folding to constrain the folding pathway of their RNA origami structures. The molecules designed by Geary et al. follow intricate folding pathways, following increasingly lower-energy states to a stable structure, and thus the pathway is not intended to visit a large number of intermediate structures, as is our design.

### Contributions of this paper

In this paper we make progress on our goal of designing RNA strands that have “long” low-barrier pathways from a given initial to a given target structure, and such that any shorter alternative pathway has a significantly higher barrier. We present a high-level overview of this design in Sect. 1.3.

With respect to a simple energy model that assigns an energy of $-1$ to each stacked pair in a structure, we prove that for any $n$, our design produces a strand of length $\varTheta (n)$ over a 4-letter alphabet whose shortest low-barrier pathway has length $\varTheta (n \log n)$. Moreover, any $o(n \log n)$ length pathway has a barrier that is at least $2 - \epsilon$ times that of the low-barrier pathway for any $\epsilon > 0$ and sufficiently large $n$.

We first design a polymer over an 8-letter alphabet, with the letters forming four distinct complementary base pairs, each of which has energy $-1$. Details of our 8-letter design and proofs of correctness with respect to the simple base pair energy model are in Sect. 2. We present our 4-letter design for the stacked pair model in Sect. 3. To improve the flow of ideas in Sects. 2 and 3, we have put some technical details in an “Appendix”. In Sect. 4, we argue heuristically that our designed pathway will be followed with high probability.

We work with a simple energy model because we want to provide rigorous proofs that our design avoids subtle unintended interactions between sub-strands, that would yield a short, low-barrier pathway. Indeed, in the process of building the proof we uncovered and fixed several design flaws, leading us to appreciate the value of a simple model. State-of-the-art energy models have thousands of parameters, and rigorous proofs for such models would be prohibitively complicated. Our proofs also do not address pseudoknot formation, or formation of base pairs between multiple copies of our designed strand, which would also compromise the design in an experimental setting. In Sect. 5, we discuss how weaknesses of our design with respect to more realistic models might be addressed in follow-on work. Conclusions are in Sect. 6.

To summarize, this work provides the first scalable designs of “long” folding pathways for single-stranded molecules. Our design does not “compute”, per se, but suggests that computations might be possible within single strands, where the number of steps of the computation grows superlinearly with the strand length. But our design ingredients may be useful in guiding the design of real RNA strands with interesting folding pathways, and can help lay the foundations for performing simple computations with single-stranded nucleic acid molecules.

### Design overview

The high-level idea underlying our design is to simulate nested loops: an outer loop with $B$ iterations and an inner loop with $A$ iterations. Each of the $A$ iterations involves a change of $\varTheta (k)$ base pairs. The total pathway length is thus $\varTheta (ABk)$ (summing over $AB$ iterations, each of length $\varTheta (k)$), while the total sequence length is only $\varTheta ((A+B)k)$.

**Fig. 1:**
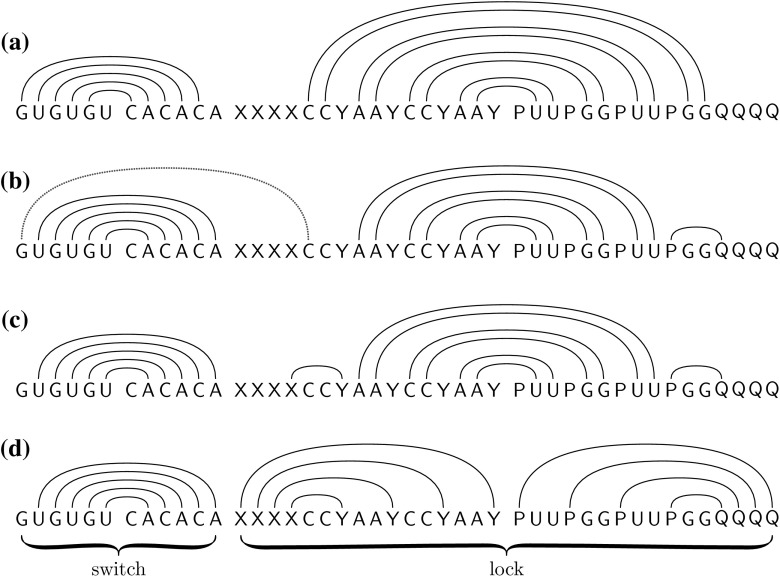
The sequence $${\text {switch-lock}}(k,A,B)$$ with $$A=6 $$ and $$B=4 $$, and some minimum free energy structures along our designed pathway from initial structure to target structure. Each letter represents $$ k $$ bases, and each *arc* represents a band of $$ k $$ base pairs between complementary bases. The pathway models the execution of a nested loop with $$A$$ outer iterations and $$B$$ inner iterations, with each iteration involving $$\varTheta (k)$$ pathway steps (arc additions and removals). **a** Initial structure. The switch, in its *left position*, has $$A-1 = 5$$ bands, leaving a region of unpaired $${\mathsf {A}}$$’s at its right end and a region of unpaired $${\mathsf {U}}$$’s at its center. The lock has $$B=4$$ bands, two $${\mathsf {C}}\cdot {\mathsf {G}}$$ bands and two $${\mathsf {A}}\cdot {\mathsf {U}}$$ bands. **b** Intermediate structure, with outermost band of the initial lock structure removed. The switch has changed to its *right position* via a barrier-$$(k+1)$$ pathway of $$\varTheta (Ak)$$ arc removals and additions (not shown). Facilitated by this switch change, *trans-arcs* (*dotted red arcs*) are possible between the $$ k $$ leftmost $${\mathsf {G}}$$’s of the switch and $${\mathsf {C}}$$’s of the lock. **c** Intermediate structure, marking the end of the first iteration of the outer loop. The trans-arcs have been removed and an $${\mathsf {X}}\cdot {\mathsf {Y}}$$ band has been added. **d** Final (target) structure obtained by successive iterations of the outer loop, the $${\mathsf {A}}\cdot {\mathsf {U}}$$, $${\mathsf {C}}\cdot {\mathsf {G}}$$ and $${\mathsf {A}}\cdot {\mathsf {U}}$$ bands of the lock are removed from the outside in, and replaced by $${\mathsf {X}}\cdot {\mathsf {Y}}$$ and $${\mathsf {P}}\cdot {\mathsf {Q}}$$ bands (details not shown). The outer iterations require that the switch alternernate between *left position* *right positions*, with each alternation corresponding to an iteration of the inner loop

Our 8-letter design is the concatenation of a lock and a switch, which embody the outer and inner loops respectively. See Fig. 1 for an illustration when the number of outer loop iterations $$B$$ is 4 and the number of inner loop iterations $$A$$ is 6. Part (a) shows the initial structure: the lock has $$B$$ “bands”, or stems, with $$2 k $$ bases each, connecting the left part of the lock to the right part of the lock (center arcs). Part (d) shows the target structure, where the lock has $$2 B $$ bands with $$ k $$ bases each (connecting $$\mathsf {X}$$ bases to $$\mathsf {Y}$$ bases and $$\mathsf {P}$$ bases to $$\mathsf {Q}$$ bases). Without the switch, any folding pathway from the initial to target lock structure necessarily has a barrier of $$2 k $$, because one band of $$2 k $$ center arcs from the initial structure must be removed to add two bands (one band of $$ k $$ $$\mathsf {X}\cdot \mathsf {Y}$$-arcs and one band of $$ k $$ $$\mathsf {P}\cdot \mathsf {Q}$$-arcs) of the target.

However, if bases of the lock pair with bases at the ends of the switch, forming *trans-arcs* as illustrated by the dotted red arcs in part (b) of the figure, the barrier can be reduced to $k+1$. To make room for the trans-arcs, the switch must first change from its *left position* structure of part (a) to its *right position* structure, illustrated in part (b). This requires the removal of $A$ bands of the switch and the addition of $A$ new bands. These removals and additions correspond to $A$ iterations of an inner loop, with each iteration involving $\varTheta (k)$ pathway steps (arc removal and additions), since $k$ is the number of arcs in a band of the switch.

Thus in the overall barrier-$(k+1)$ folding pathway, the lock alternately forms base pairs with left and right ends of the switch, $B$ times in total; upon each alternation the switch shifts from left to right position or vice versa via $A$ band removals and additions. Each band removal and addition requires $\varTheta (k)$ arc removals and additions, for a total pathway length of $\varTheta (ABk)$.

By varying the number of bands $B$ in the initial lock structure, the number of bands $A$ in the initial switch structure, and the number $k$ of bases in initial bands of the switch and lock, we obtain different tradeoffs between the length of the low-barrier pathway and the gap between this low barrier and the higher barrier of shorter alternatives.

To get from the 8-letter design to a four letter design, we need to map our ${\mathsf {X}}$, ${\mathsf {Y}}$, ${\mathsf {P}}$, and ${\mathsf {Q}}$ sequences to sequences over $\{{\mathsf {A}}, {\mathsf {C}}, {\mathsf {G}}, {\mathsf {U}}\}$ so that ${\mathsf {X}}$ is complementary to ${\mathsf {Y}}$, ${\mathsf {P}}$ is complementary to ${\mathsf {Q}}$, and no other pair of band sequences involving at least one ${\mathsf {X}}$, ${\mathsf {Y}}$, ${\mathsf {P}}$, or ${\mathsf {Q}}$ sequence will stably bind to each other in the stacked pair model. We achieve this by choosing the ${\mathsf {X}}$, ${\mathsf {Y}}$, ${\mathsf {P}}$, and ${\mathsf {Q}}$ to have alternating symbols, e.g., ${\mathsf {X}}^{4} = {\mathsf {C}}{\mathsf {A}}{\mathsf {C}}{\mathsf {A}}$, etc.

## The 8-letter alphabet design

In this section we will present our polymer design over an 8-letter alphabet. While it is possible to use synthetic nucleic acids to realize this polymer, the main reason why we are doing this is that it allows us to simplify the construction. In the next section, we will then map this construction to a 4-letter alphabet, and prove that it retains the desired properties, although the bounds will become weaker.

We first introduce notation and our designed sequence over the 8-letter alphabet in Sect. 2.2, where the designed sequence is specified as a switch whose initial structure has $$A$$ “bands” with $$ k $$ base pairs in each band, concatenated with a lock whose initial structure has $$B$$ bands with $$2 k $$ base pairs in each band. We use the terms “base pair” and “arc” interchangeably, since arcs correspond to base pairs in an arc diagram representation of a secondary structure (see Fig. 1). Then in Sect. 2.3, we bound the total number of arcs in structures with trans-arcs, i.e., arcs from the lock to the switch. In Sect. 2.4 we will use these bounds to limit occurrences of certain types of (off-center) base pairs in structures within the barrier of $$2 k - 1$$. Finally, in Sect. 2.5, we show that any pathway between the initial and the target structure with all intermediate structures within a barrier of $$2 k - 1$$ has to go through $$B$$ “milestone” structures in a fixed order. The crucial property of these milestone structures is that any two consecutive structures have the switch in different positions, i.e., a complete reconfiguration of switch is required to move from one milestone to another, from which it will follow that the length of the pathway is in $$\varOmega (kAB)$$.

### Definitions

We will use the following 8-letter alphabet: $\{\mathsf {A,C,G,U,P,Q,X,Y}\}$, where the following pairs of letters are complementary $(\mathsf {A,U})$, $(\mathsf {C,G})$, $(\mathsf {P,Q})$ and $(\mathsf {X,Y})$. We assume that only the complementary bases can form base pairs (arcs). Throughout we consider only pseudoknot-free secondary structures, i.e., structures with no crossing arcs. We will use the simple arc counting energy model, in which each base pair contributes energy $-1$ to the total energy and there are no other contributions to the total energy. Let ${\text {AC}}(S)$ denote the arc count of a secondary structure $S$ and let ${\text {MAC}}{}(s)$ denote the maximum arc count over all structures for a sequence $s$. With respect to the arc counting energy model, structures for a sequence $s$ with ${\text {AC}}$ equal to ${\text {MAC}}{}(s)$ will be referred to as minimum free energy (MFE) structures for $s$.

For a given sequence, a folding pathway is a sequence of seondary structures for that sequence, where each structure (except for the first one in the sequence) differs from its predecessor by exactly one base pair. Consider a pathway $P = S_{1},S_{2},\dots ,S_{m}$ for a sequence $s$. The barrier of $P$ is defined as the biggest gap between a low energy point and a subsequent high energy point in the pathway. In particular, if using the arc counting energy model, the *barrier* of $P$ is defined as $\max \limits _{1\le i\le j\le m} [{\text {AC}}(S_{i}) - {\text {AC}}(S_{j})]$. Note that if the initial structure of $P$ is MFE then the barrier of $P$ is simply ${\text {MAC}}{}(s) - \min \limits _{1\le j\le m} {\text {AC}}(S_{j})$.

### The design and notations

Let $k$, $A$ and $B\in \mathbb {N}$, and let $A$ and $B$ be even. The variable $A$ is distinguished from the base $\mathsf {A}$ by font. Consider the family of sequences obtained from the following regular expressions by concatenating the switch and lock sequences:

$$\begin{aligned} \begin{array}{ll} &{}\hbox {Switch }({\mathsf {G}}^k{\mathsf {U}}^k)^{A/2}({\mathsf {C}}^k{\mathsf {A}}^k)^{A/2} \\ &{}\hbox {Lock } ({\mathsf {X}}^{k})^B({\mathsf {C}}^{2k}{\mathsf {Y}}^{k}{\mathsf {A}}^{2k}{\mathsf {Y}}^{k})^{B/2}({\mathsf {P}}^k{\mathsf {U}}^{2k}{\mathsf {P}}^k{\mathsf {G}}^{2k})^{B/2}({\mathsf {Q}}^k)^B\end{array} \end{aligned}$$

For example, if $A=6$ and $B=4$, then we have the switch sequence

$$\begin{aligned} {\mathsf {G}}^k {\mathsf {U}}^k {\mathsf {G}}^k {\mathsf {U}}^k {\mathsf {G}}^k {\mathsf {U}}^k {\mathsf {C}}^k {\mathsf {A}}^k {\mathsf {C}}^k {\mathsf {A}}^k {\mathsf {C}}^k {\mathsf {A}}^k \end{aligned}$$

and the lock sequence

$$\begin{aligned}&{\mathsf {X}}^{k}{\mathsf {X}}^{k}{\mathsf {X}}^{k}{\mathsf {X}}^{k}{\mathsf {C}}^{2k}{\mathsf {Y}}^{k}{\mathsf {A}}^{2k}{\mathsf {Y}}^{k}{\mathsf {C}}^{2k}{\mathsf {Y}}^{k}{\mathsf {A}}^{2k}{\mathsf {Y}}^{k} {\mathsf {P}}^k{\mathsf {U}}^{2k}{\mathsf {P}}^k{\mathsf {G}}^{2k}{\mathsf {P}}^k{\mathsf {U}}^{2k}\\&\quad {\mathsf {P}}^k{\mathsf {G}}^{2k}{\mathsf {Q}}^k{\mathsf {Q}}^k{\mathsf {Q}}^k{\mathsf {Q}}^k \end{aligned}$$

We denote the switch sequence by ${\text {switch}}(k,A)$, the lock sequence ${\text {lock}}(k, B)$, and the concatenated switch-lock sequence by ${\text {switch-lock}}(k,A,B)$; its length is $n = 2kA+ 8kB$.

For convenience, we will refer to the $\mathsf {G}$ and $\mathsf {U}$ portion of the switch as the left side, and the $\mathsf {C}$ and $\mathsf {A}$ portion as the right side. Similarly, the left side of the lock is the sequence containing letters $\mathsf {X}$, $\mathsf {Y}$, $\mathsf {C}$, and $\mathsf {A}$ and the right side of the lock contains $\mathsf {P}$, $\mathsf {Q}$, $\mathsf {U}$, and $\mathsf {G}$.

Let a *region* be the maximal substring of consecutive identical bases. We label the regions of the switch and lock as follows:

$$\begin{aligned} \begin{array}{ll} &{}\underbrace{\mathsf {G}^{k}}_{L_{1}} \underbrace{\mathsf {U}^{k}}_{L_{2}} \dots \underbrace{\mathsf {G}^{k}}_{L_{A- 1}} \underbrace{\mathsf {U}^{k}}_{L_{A}} \underbrace{\mathsf {C}^{k}}_{R_{A}} \underbrace{\mathsf {A}^{k}}_{R_{A- 1}} \dots \underbrace{\mathsf {C}^{k}}_{R_{2}} \underbrace{\mathsf {A}^{k}}_{R_{1}}\\ &{}\underbrace{\mathsf {X}^{k}}_{x_{B}} \dots \underbrace{\mathsf {X}^{k}}_{x_{1}} \underbrace{\mathsf {C}^{2k}}_{l_{1}} \underbrace{\mathsf {Y}^{k}}_{y_{1}} \dots \underbrace{\mathsf {A}^{2k}}_{l_{B}} \underbrace{\mathsf {Y}^{k}}_{y_{B}} \underbrace{\mathsf {P}^{k}}_{p_{B}} \underbrace{\mathsf {U}^{2k}}_{r_{B}} \dots \underbrace{\mathsf {P}^{k}}_{p_{1}} \underbrace{\mathsf {G}^{2k}}_{r_{1}} \underbrace{\mathsf {Q}^{k}}_{q_{1}} \dots \underbrace{\mathsf {Q}^{k}}_{q_{B}} \end{array} \end{aligned}$$

Figure 1 depicts our initial and target structures above and below sequence $${\text {switch-lock}}(k,A,B)$$. The initial structure contains $$ k $$ arcs between regions $$L_i$$ and $$R_{i+1}$$ of the switch, for $$1 \le i \le A- 1$$ and $$2 k $$ arcs between regions $$l_i$$ and $$r_i$$ of the lock, for $$1 \le i \le B$$. The target structure contains $$ k $$ arcs between regions $$L_{i+1}$$ and region $$R_i$$ of the switch, for $$1 \le i \le A- 1$$, and $$ k $$ arcs between regions $$x_i$$ and $$y_i$$ of the lock plus $$ k $$ arcs between regions $$p_i$$ and $$q_i$$ of the lock, for $$1 \le i \le B$$. We sometimes refer to the set of arcs between two regions as *bands*.

**Fig. 2:**
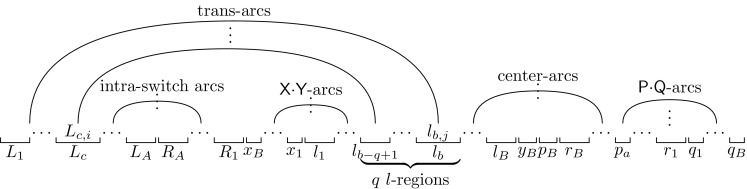
Notation used when considering left–left trans-arcs

We denote the $i$-th *leftmost* base in region $L_{a}$ ($l_{a}$) as $L_{a,i}$ ($l_{a,i}$) and the $i$-th *rightmost* base in region $R_{a}$ ($r_{a}$) as $R_{a,i}$ ($r_{a,i}$).

Let $u$ and $v$ be two regions. When we refer to a “$u\cdot v$-arc”, we mean any arc from a base in region $u$ to a base in region $v$. This is only possible if the two bases are complementary. We sometimes refer to such an arc as a “$u$-arc” if it is not important what is the region $v$. In particular, $l_a\cdot r_b$-arcs will be called *center-arcs*—see Fig. 2 for an illustration of center-arcs and other types of arcs introduced next. A center-arc is *off-center* if $a \ne b$ and is *on-center* otherwise. (All center-arcs in Fig. 1 are on-center.) We refer to arcs between the switch and lock as *trans-arcs*. Note that all arcs are either *intra-switch*, *intra-lock* or trans-arcs. We say that an intra-switch arc from region $L_{a}$ to region $R_{b}$ is in the *left (right) position* if $b = a + 1$ ($b = a - 1$). In the initial structure, all intra-switch arcs are in the left position, and in the target structure all intra-switch arcs are in the right position—see Fig. 1.

We have several claims that establish the minimum free energy (MFE) structures for the sequences of the switch, lock, and switch and lock. The proofs and the auxiliary claims used to establish these facts are given in the “Appendix”.

#### Claim 1

All MFE structures of ${\text {switch}}(k,A)$ have ${\text {MAC}}{}_{\mathrm {switch}}(k,A){}:= (A- 1)k$ arcs.

#### Claim 2

All MFE structures of ${\text {lock}}(k, B)$ have ${\text {MAC}}{}_{\mathrm {lock}}(k,B){}:= 2k B$ arcs.

#### Claim 3

All MFE structures of the switch and lock sequence ${\text {switch-lock}}(k,A, B)$ have ${\text {MAC}}{}(k,A, B){}:= {\text {MAC}}{}_{\mathrm {switch}}(k,A){}+ {\text {MAC}}{}_{\mathrm {lock}}(k,B){}$ arcs. The initial and target structures are MFE structures.

To conclude this section, we describe our long, low-barrier folding pathway from the initial to target structure of ${\text {switch-lock}}(k,A, B)$.

#### Claim 4

Let $B$ be even. There is a pathway from the initial to the target structure of ${\text {switch-lock}}(k,A, B)$ with barrier $k + 1$ and with length $2k(AB+ A+ 2B- 1)$.

#### Proof

We first describe a folding pathway that causes the switch to be reconfigured from the initial switch structure (all intra-swich arcs are in the left position) to the target switch structure (all intra-swich arcs are in the right position)—see the left side of Fig. 1 for an illustration of these structures. Note that it is necessary to remove all arcs of the initial switch structure and add all of the arcs of the target, since they have no arcs in common. This can be done in $2k(A- 1)$ steps with barrier $k + 1$ as follows, in three subphases:

- *Barrier*-$k$ *ascent* Remove all $k$ $L_{1}\cdot R_{2}$-arcs.
- *Branch migration* Repeatedly remove the outermost arc in the left position, say an $L_{\sigma }\cdot R_{\sigma + 1}$-arc, and immediately add an arc between the freed base in $L_{\sigma }$ and the rightmost available base in $R_{\sigma - 1 }$.
- *Barrier*-$k$ *descent* Add the $k$ innermost arcs of the target switch structure.

Note that the reverse of this folding pathway, with arc additions replaced by arc removals and vice versa, reconfigures the switch from its target to initial structure.

We next describe a folding pathway that “unlocks” the $i$-th band of the initial lock structure, i.e., removes the band between regions $l_i$ and $r_i$. We consider the case where $i$ is odd; the pathway when $i$ is even is similar. First, reconfigure the switch from the left to the right position as described above; this exposes the outermost $\mathsf {G}$ at the left of the switch. Then:

- *Branch migration* Repeatedly, for $k$ iterations, remove the outermost $l_{i}\cdot r_{i}$-arc and add a trans-arc from the newly freed $\mathsf {C}$ in the lock to the leftmost free $\mathsf {G}$ in the leftmost region of the switch.
- *Barrier*-$k$ *ascent* Remove the remaining $k$ $l_{i}\cdot r_{i}$-arcs.
- *Barrier*-$k$ *descent* Form $k$ $p_{i}\cdot q_{i}$-arcs.
- *Barrier*-$k$ *ascent* Remove the trans-arcs added in the branch migration step above.
- *Barrier*-$k$ *descent* Form $k$ $x_{i}\cdot y_{i}$-arcs.

This pathway has barrier $$k + 1$$ and requires $$6 k $$ steps plus the steps to switch the switch, i.e., $$2kA+ 4k$$ steps. This is repeated $$B$$ times.

Finally, we need to reconfigure the switch one more time, so that it is in the target position. Hence, the total number of steps of this pathway from the initial to the target structure is $2k(A+ 2)B+ 2k (A- 1)$. $\square$

### Bounding the arc count in structures with trans-arcs

We want to obtain upper bounds on the number of arcs in structures with trans-arcs, i.e., arcs from a base in the switch to a base in the lock. This will be useful, because we can conclude that many such structures, e.g., structures with too many trans-arcs, cannot be on low-barrier pathways.

Each trans-arc either connects the left part of the switch with the left part of the lock, or the right part of the switch with the right part of the lock. In addition, all left–left trans-arcs cross all right–right trans-arcs, therefore each structure can contain only one type of trans-arcs.

For any structure with trans-arcs, we will use the following notation throughout this subsection. First, suppose that there are trans-arcs from the left part of the switch to the left part of the lock. Let $c$ be the largest number such that switch region $L_{c}$ is involved in trans-arcs. Assume that the outermost trans-arc pairs with lock base $l_{b,j}$ and the innermost trans-arc pairs $L_{c,i}$ with a base in the region $l_{b- q+ 1}$, i.e., $q$ is the number of lock regions that can only be involved in trans-arcs. Let the outermost $\mathsf {P}\cdot \mathsf {Q}$-arc have an endpoint in region $p_a$. Let $t$ be the number of trans-arcs involving switch region $L_{1}$.

Next, suppose that there are trans-arcs from the right part of the switch to the right part of the lock. In this case, symmetric to the left case above, let $c$ be the largest number such that switch region $R_{c}$ is involved in trans-arcs. Assume that the innermost trans-arc pairs with lock base $r_{b,j}$ and the outermost trans-arc pairs $R_{c,i}$ with a base in the region $l_{b- q+ 1}$. Let the outermost $\mathsf {X}\cdot \mathsf {Y}$-arc have an endpoint in region $y_a$. Let $t$ be the number of trans-arcs involving switch region $R_{1}$.

Finally, in both cases (left–left trans-arcs and right–right trans-arcs), let $T$ be the number of trans-arcs. Figure 2 illustrates these definitions in the left–left case. We will use these quantities to bound the number of different types of arcs in a structure. We provide one such bound here; several others are in the “Appendix”. The following claim shows that ${\text {AC}}(S)$ must be “low” for structures $S$ with trans-arcs that connect regions that are not close to the outside of the switch (i.e., $c + q > 4$).

#### Claim 5

Consider a structure $S$ for ${\text {switch-lock}}(k,A, B)$, where $S$ has trans-arcs. Suppose that $c+ q> 4$. Then ${\text {AC}}(S)\le {\text {MAC}}{}(k,A, B){}- 2k$.

### Bounding the arc count in structures with off-center arcs

Recall on-center and off-center arcs, which we defined in Sect. 2.2. The next claim limits occurrences of off-center arcs in structures with at least ${\text {MAC}}{}(k,A, B){}- 2k$ arcs.

#### Claim 6

Let $S$ be a structure for ${\text {switch-lock}}(k,A, B)$, in which an on-center arc covers an off-center arc. Then ${\text {AC}}(S)\le {\text {MAC}}{}(k,A, B){}- 2k$.

It follows that in any structure $S$ with ${\text {AC}}(S) > {\text {MAC}}{}(k,A, B){}- 2k$, all arcs covered by an on-center arc are also on-center arcs. We have the following corollary.

#### Corollary 1

Let $S$ be a structure for *switch and lock sequence* ${\text {switch-lock}}(k,A, B)$ *with* ${\text {AC}}(S) > {\text {MAC}}{}(k,A, B){} - 2k$, *such that* $S$ *has an on-center arc* $\alpha$ *between regions* $l_\sigma$ *and* $r_\sigma$ *of the lock. Then for every* $\sigma ' , \sigma < \sigma ' \le B$, *there is at least one on-center arc from lock region* $l_{\sigma '}$ *to lock region* $r_{\sigma ' }$.

### The main proof

Consider a pathway $P = S_{1},S_{2},\dots$ from the initial to the target structure. Let $p_i$ be the index of the first structure of pathway $P$ that has no on-center arc from region $l_{i}$ to region $r_{i}$ of the lock and such that no subsequent structures of $P$ have such an arc either.

#### Claim 7

If pathway $P$ from the initial structure to the target structure of ${\text {switch-lock}}(k,A, B)$ has barrier at most $2k - 1$, $P$ must remove on-center arcs from the outside in, i.e., $p_1< p_2< \dots < p_{B}$.

#### Proof

Assume to the contrary that $p_{i + 1} \le p_{i}$ for some $i$. Consider structure $S_{p_{i} - 1}$ of pathway $P$. By the definition of $p_{i}$, $S_{p_{i} - 1}$ must contain an $l_{i}\cdot r_{i}$-arc. By Corollary 1, $S_{p_{i} - 1}$ contains also an $l_{i + 1}\cdot r_{i + 1}$-arc. Since $S_{p_{i}}$ removes the $l_{i}\cdot r_{i}$-arcs, it still contains the $l_{i + 1}\cdot r_{i + 1}$-arc, which contradicts the fact that $p_{i + 1} \le p_{i}$. $\square$

#### Claim 8

Suppose that $i$ is such that ${\text {AC}}(S_{p_{i}}) > {\text {MAC}}{}(k,A, B){}- 2k$. If $i$ is odd, then all intra-switch arcs must be in the right position and if $i$ is even, all intra-switch arcs must be in the left position.

#### Corollary 2

*If* ${\text {AC}}(S_{p_{i} - 1}),{\text {AC}}(S_{p_{i}}) ,{\text {AC}}(S_{p_{i + 1} - 1}),{\text {AC}}(S_{p_{i + 1}})> {\text {MAC}}{}(k,A, B){}- 2k$, *the number of steps (i.e., structures in the pathway* $P$) *from* $S_{p_i}$ *to* $S_{p_{i+1}}$ *is at least* $2(A- 4)k$.

**Fig. 3:**
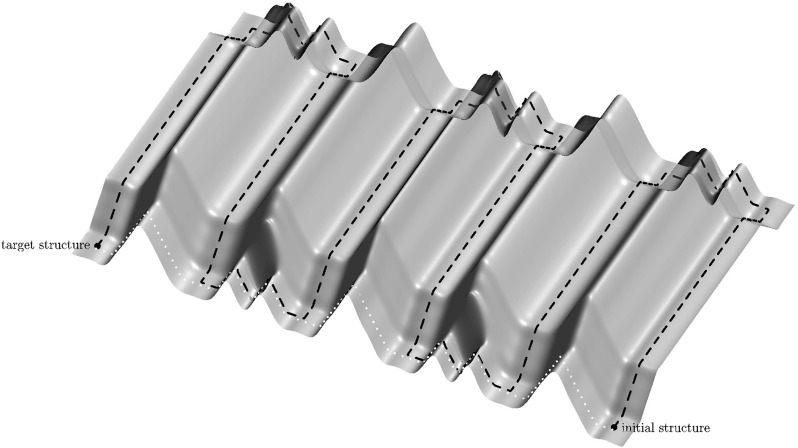
Illustration of the long low-barrier and the short high-barrier pathways from the initial to the target configurations of the switch and lock sequence. The long pathway is depicted with a *black dashed line*: vertical long stretches correspond to switching the switch from one position to another, and two “hills” at the ends correspond to unlocking one band of the lock. The short pathway depicted with a white *dotted line* along the bottom border avoids switching the switch, but needs to ascend over a higher peak when unlocking every other band of the lock

We are now ready to prove the main result of this section, namely that to avoid a high barrier along a pathway from initial to target structure, it is necessary to follow a long pathway. Figure 3 illustrates the difference between the long low-barrier and the short high-barrier pathways from the initial to the target configurations of the switch and lock sequence.

#### Theorem 1

*Let* $B$ *be even. There is a pathway from the initial to the target structure of* ${\text {switch-lock}}(k,A, B)$ *with barrier* $k + 1$ *and with length* $2k(AB+ A+ 2B- 1)$. *Moreover, any pathway from the initial to the target structure with barrier at most* $2k - 1$ *has length at least* $2k(AB- A- 4B+ 4)$.

#### Proof

The first part of the theorem follows from Claim 4. The second part of the theorem follows by Claim 7 and Corollary 2. $\square$

#### Corollary 3

*For any constant* $C>0$, *for any positive integer* $n$, *there is a sequence of length* $n + O(\log n)$ *over the 8-letter alphabet with two MFE structures such that the shortest pathway between these structures with barrier between* $C\log {n} + 1$ *and* $2C\log {n} - 1$ *has length* $\varTheta (n^{2}/\log {n})$.

#### Proof

Set $k$ to be $\lceil C\log n \rceil$, $B$ to be the smallest even number that is greater than or equal to $\frac{n}{16 k}$, and $A$ to be $4B$. Then the length of sequence ${\text {switch-lock}}(k,A, B)$, which is $2kA + 8kB$, is $n + O(\log n)$. By Theorem 1, ${\text {switch-lock}}(k,A, B)$ satisfies the conditions of this corollary. $\square$

## The 4-letter alphabet design using the stacked base pair energy model

For sequences over the 4-letter alphabet, we will use a different energy model, the *stacked base pairs* energy model. Stacked base pairs, or stacked arc pairs, are two consecutive base pairs, one between positions $i$ and $j$ and the second between positions $i + 1$ and $j - 1$ of the sequence, for some $i$ and $j$. In our energy model, each stacked arc pair contributes the same energy ($-1$) and there are no other contributions to the total free energy of the structure. In a more realistic energy model, the energy of each stacked arc pair would depend on the bases. Let ${\text {SAC}}(S)$ denote the number of stacked arc pairs of a structure $S$ and ${\text {MSAC}}{}(s)$ the maximum number of stacked arc pairs over all structures for a sequence $s$.

### General results

Let $s$ be a sequence over the 8-letter alphabet $\{\mathsf {A,C,G,U,P,Q,X,Y}\}$ of length $n$ with $r$ regions. Map each $u$-region $u^{m}$, where $u\in \{\mathsf {A}, \mathsf {C}, \mathsf {G}, \mathsf {U}\}$, to a sequence $u^{m + 1}$, and each $u$-region $u^{m}$, where $u\in \{\mathsf {P}, \mathsf {Q}, \mathsf {X}, \mathsf {Y}\}$, to a sequence of length $m + 1$ of alternating $\mathsf {AG}$’s, $\mathsf {UC}$’s, $\mathsf {CA}$’s, $\mathsf {GU}$’s, respectively. We will still refer to these subsequences as $u$-regions. The new sequence $s' = {\text {map}}(s)$ is a sequence over the 4-letter alphabet $\{\mathsf {A}, \mathsf {C}, \mathsf {G}, \mathsf {U}\}$ of length $n + r$.

Let $s$ be a sequence over the 8-letter alphabet. Consider any structure $S$ for the 4-letter sequence $s' = {\text {map}}(s)$. We say that an arc of $S$ is *eccentric* if it connects a $u$-region to a $v$-region, where $u$ and $v$ are not complementary in the 8-letter alphabet. For instance, an arc connecting any base of a $\mathsf {G}$-region and any base $\mathsf {C}$ of an $\mathsf {X}$-region is eccentric. A stacked arc pair between positions $i$ and $j$, and $i + 1$ and $j - 1$, is called *eccentric* if (a) either $i$ and $i + 1$ or $j - 1$ and $j$ belong to different regions, or (b) one of its arcs is eccentric.

We refer to the first and last bases of each region that is adjacent to another region as *boundary bases*. A *boundary* is the pair of neighbouring boundary bases. To bound the number of eccentric stacked arc pairs, we observe the following.

#### Claim 9

Let $$ s $$ be a sequence over the 8-letter alphabet. Let $$ S '$$ be a structure for $$s' = {\text {map}}(s)$$. For any eccentric stacked arc pair of $$ S '$$ between positions $$ i $$ and $$ j $$, and $$i + 1$$ and $$j - 1$$, either pair $$i,i + 1$$ or pair $$j - 1,j$$ is a boundary.

#### Corollary 4

*Let* $s$ *be a sequence over the 8-letter alphabet with* $r$ *regions. Let* $S'$ *be a structure for* $s' = {\text {map}}(s)$. *The total number of eccentric stacked arc pairs in* $S'$ *is at most* $r - 1$.

#### Proof

The number of the boundaries in $s'$ is the number of all regions in the sequence minus 1, i.e., $r - 1$. Since each boundary can be used by at most one stacked arc pair, it follows that the number of eccentric stacked arc pairs is at most $r - 1$. $\square$

The next claim shows how to convert a structure $S'$ of $s' = {\text {map}}(s)$ to a structure for $s$ with the same number of arcs as the number of non-eccentric stacked arc pairs of $S'$. For this we need to define a new mapping from structures of $s' = {\text {map}}(s)$ over the 4-letter alphabet to structures of $s$ over the 8-letter alphabet. Let $S'$ be a structure for $s'$. Then we define $S ={\mathrm{Map}^{\prime }}(S')$ as follows. For each non-eccentric stacked arc pair in $S'$, let the outer arc of the two stacked arcs connect the $i$-th position of a region $u$ and the $j$-th position of a region $v$. We add an arc to $S$ connecting the $i$-th base of the region $u$ and the $(j - 1)$-th base of $v$. Note that $j > 1$, since the right end of the inner arc of the stacked arc pair lies in the same region $v$.

#### Claim 10

Let $s$ be a sequence over the 8-letter alphabet. Let $S'$ be a structure for $s' = {\text {map}}(s)$ with $E$ eccentric arc pairs. Then $S = {\mathrm{Map}^{\prime }}(S')$ is a structure for $s$ with ${\text {AC}}(S) = {\text {SAC}}(S') - E$.

#### Proof

The claim follows directly from the definition of ${\mathrm{Map}^{\prime }}()$.

We can now prove the main theorem which extends the claim about the length of pathways between two structures for a sequence $s$ over the 8-letter alphabet to a similar claim for the 4-letter sequence $s' = {\text {map}}(s)$.

#### Theorem 2

*Let* $$ s $$ *be a sequence over the 8-letter alphabet and let* $$s' = {\text {map}}(s)$$. *Assume that any structure for* $$s'$$ *with at least* $${\text {MAC}}{}(s) - (K - E)$$ *stacked arc pairs has at most* $$ E $$ *eccentric stacked arc pairs. Let* $$S_1'$$ *and* $$S_2'$$ *be two structures of* $$s'$$, *and let* $$S_1 = {\mathrm{Map}^{\prime }}(S_1')$$ *and* $$S_2 = {\mathrm{Map}^{\prime }}(S_2')$$ *be structures of* $$ s $$. *Let* $$D = {\text {SAC}}(S_{1}') - {\text {MAC}}{}(s)$$. *Suppose that any pathway between* $$S_{1}$$ *and* $$S_{2}$$ *with barrier at most* $$ K $$ *has length at least* $$ L $$. *Then any pathway between* $$S_1'$$ *and* $$S_2'$$ *with barrier at most* $$K + D - E$$ *has length at least* $$ L /2$$.

We can apply this theorem to the sequence $s = {\text {switch-lock}}(k,A, B)$. Define the initial and target structures for $s' = {\text {map}}(s)$ in the natural way, to be those structures that map to the initial and target structures for $s$ under the mapping ${\mathrm{Map}^{\prime }}()$. Then we have:

#### Corollary 5

Let $s' = {\text {map}}({\text {switch-lock}}(k,A, B))$, where *B* is even. *Then there exists a pathway from the initial to the target structure of* $s'$ *with barrier* $k + 3$ *and with length* $(2(k + 1)(A+ 2) - 3)B+2(k + 1)(A- 1)$. *The length of any pathway from the initial to the target structure with barrier at most* $2k - A- 6B- 2$ *is at least* $k(A- 4)(B- 1)$.

The second part of Corollary 5 is useful only if $A+ 6 B< k$. In this case the barrier is in $\varOmega (\sqrt{n})$, where $n$ is the length of the sequence. It is more practical to have a barrier logarithmic in the length of the sequence which we will achieve in the following subsection.

### The switch and lock sequence for the 4-letter alphabet

In this section we will improve the result of Corollary 5 by showing that the bounds on the barrier of a long pathway from the initial to the target structure of the switch and lock depend on $B$, but not on $A$. To achieve this we will include eccentric intra-switch stacked arc pairs in our design (thus, we will need to amend the definition of eccentric stacked arc pairs to exclude these arc pairs). We also need to modify slightly the mapping ${\text {map}}()$ of the switch and lock sequence to the 4-letter alphabet: we will leave the sequence of the switch unchanged, while mapping the sequence of the lock as described in Sect. 3.1. We will assume that $k$ is even. The regular expressions that produce sequences for the switch and lock are as follows.

$$\begin{aligned} \begin{array}{ll} \hbox {Switch } &{} [\mathsf {G}^{k}\mathsf {U}^{k}]^{A/2} [\mathsf {C}^{k}\mathsf {A}^{k}]^{A/2} \\ \hbox {Lock } &{} [(\mathsf {CA})^{k/2}\mathsf {C}]^{B} [\mathsf {C}^{2k+1}\,(\mathsf {GU})^{k/2}\mathsf {G}\, \mathsf {A}^{2k+1}\,(\mathsf {GU})^{k/2}\mathsf {G}]^{B/2}\cdot \\ &{} [(\mathsf {AG})^{k/2}\mathsf {A}\,\mathsf {U}^{2k+1}\, (\mathsf {AG})^{k/2}\mathsf {A}\,\mathsf {G}^{2k+1}]^{B/2} [(\mathsf {UC})^{k/2}\mathsf {U}]^{B} \end{array} \end{aligned}$$

We denote the switch sequence by ${\text {switch}}^{\prime }(k,A)$, the lock sequence by ${\text {lock}}^{\prime }(k, B)$, and the concatenated sequence by ${\text {switch-lock}}^{\prime }(k,A, B)$ (its length is $2kA+ (8k+6)B$). The initial and target structures of ${\text {switch-lock}}^{\prime }(k,A, B)$ are defined analogously to the initial and target structures of ${\text {switch-lock}}(k,A, B)$, however the initial structure has $2k + 1$ arcs between regions $l_{i}$ and $r_{i}$ and the target structure has $k + 1$ arcs between regions $x_{i}$ and $y_{i}$, and regions $p_{i}$ and $q_{i}$. Let ${\text {MSAC}}(k,A, B){}$ denote the maximum number of stacked arc pairs over all structures for ${\text {switch-lock}}^{\prime }(k,A, B)$.

We will use the same definition of eccentric arcs as above, however, a stacked arc pair between positions $i$ and $j$, and $i + 1$ and $j - 1$ is called *eccentric* if at least one of its arcs is eccentric. The difference between this new definition and the definition from the previous section is that the intra-switch stacked arc pairs that connect a boundary to a boundary are not considered eccentric. Note however, that the boundaries of the switch can be still involved in eccentric stacked arc pairs if they are composed of trans-arcs.

Theorem 2 relies on Claim 10, so we need to prove a new variant of that claim for our new sequence ${\text {switch-lock}}^{\prime }(k,A, B)$. To redefine map ${\mathrm{Map}^{\prime }}()$, we will use the following mapping of non-eccentric stacked arc pairs of ${\text {switch-lock}}^{\prime }(k,A, B)$ to arcs of ${\text {switch-lock}}(k,A, B)$: Consider a pair of non-eccentric stacked arcs. Let the outer arc of this pair connect the $i$-th position of a region $u$ and the $j$-th position of a region $v$. We map this pair to the arc connecting the $i$-th base of the region $u$ and the $j$-th base of the region $v$ if $v$ is in the switch or the $(j - 1)$-st base of $v$ if $v$ is in the lock. It is easy to check that Claim 10, and thus also Theorem 2 hold with these new definitions of eccentric arcs, mappings ${\text {map}}()$ and ${\mathrm{Map}^{\prime }}()$.

By Claim 9 we have that the number of eccentric stacked arc pairs is at most $2A+ 6B- 1$. We will improve this bound by showing that the number of these pairs does not depend on $A$ for structures within a specific barrier.

#### Claim 11

Consider a structure $S'$ for ${\text {lock}}^{\prime }(k, B)$. Then the number of non-eccentric stacked arc pairs in $S'$ is at most $(2k + 1)B$.

#### Claim 12

For any structure $S'$ of ${\text {switch-lock}}^{\prime }(k,A, B)$ with at least ${\text {MAC}}{}(k,A, B){}- 2k + 8B+ 4$ stacked arc pairs, the number of its eccentric stacked arc pairs is at most $8B+ 2$.

Using these results and Theorem 2, we have the following result.

#### Theorem 3

*Consider the sequence* ${\text {switch-lock}}^{\prime }(k,A, B)$. *There is a pathway from the initial to the target structure with barrier* $k + 2$ *and with length* $(2k(A- 1) + 6k + 3)B+2k(A- 1 )$. *Moreover, any pathway from the initial to the target structure of* ${\text {switch-lock}}^{\prime }(k,A, B)$ *with barrier at most* $2k - 8B- 5$ *has length at least* $k(A- 4)(B- 1) - 1$.

#### Corollary 6

*For any constants* $C>0$ *and* $\epsilon >0$, *for any positive integer* $n$, *there is a sequence of length* $n + \varTheta (\log ^2 n)$ *over the 4-letter alphabet with two structures such that the shortest pathway between these two structures with barrier between* $C \log n + O(1)$ *and* $(2-\epsilon )C\log n - O(1)$ *has length* $\varTheta (n\log n)$, *where the constant hidden in this* $\varTheta$ *depends linearly on* $C$ *and* $\epsilon$.

#### Proof

Set $$ k $$ to be $$\lceil C\log n \rceil $$, $$A$$ to be the smallest even number that is greater than or equal to $$ n /2k$$, and $$B$$ to be $$\lceil k\epsilon /8 \rceil $$. The result follows immediately from Theorem 3.

Corollary 6 implies that for any $$\epsilon >0$$, for sufficiently large $$ n $$, there is a sequence of length $$n + o(n)$$ whose low-barrier folding pathways grow superlinearly in $$ n $$, and for which any significantly shorter folding pathway, say of length $$ O ( n )$$, has an energy barrier that is $$2-\epsilon $$ times that of the low-barrier pathway. To see this, substitute $$\epsilon /4$$ for $$\epsilon $$ in the statement of Corollary 6, and choose $$ n $$ large enough so that $$ O (1)$$ terms in the lower and upper bounds on the barrier are less than $$\epsilon C\log n/4$$. Then the ratio of the upper and lower barrier range endpoints, namely $$(2-\epsilon /2)C\log n - O(1)$$ divided by $$C \log n + O(1)$$, is at least $$2 - \epsilon $$.

## On the likelihood of following a low-barrier pathway

Although they establish barrier gaps, Theorems 1 and 3 do not address the following question: Which is the more likely route from an initial to target structure of our designed sequence: a low-barrier pathway that requires repeated recongifuration of the switch, and thus visits many distinct structures, or an alternative high-barrier pathway? In this section we provide a heuristic argument that for sufficiently long sequences, a low-barrier pathway is more likely to be followed.

In Sect. 4.1, we first prove exponential upper and lower bounds on the expected time, i.e., number of arc addition and removal steps, needed to ascend a barrier of size $k$, for a simple stochastic folding model. Then in Sect. 4.2 we show simulation results, indicating that the exponential bounds may hold for somewhat more complex folding models. In Sect. 4.3 we then use the upper bound of Sect. 4.1, along with some informal arguments, to bound the expected time to follow the low-barrier pathway. This time is dominated by the time to remove all $B$ center bands in the initial lock structure (and the associated time for the switch to reconfigure from its left and right orientation). Finally, in Sect. 4.4 we argue that this expected time to follow a low-barrier pathway is significantly faster than the expected time to follow an alternative, high-barrier pathway, and conclude that the low-barrier pathway is more likely to be followed.

### Bounds on scaling a barrier

We start by proving a bound on the expected time to ascend a barrier, for a stochastic folding pathway model. By ascending a barrier, we mean that $k$ initially-present arcs are removed, where the only re-pairing of bases that form the arcs is with other bases within the arcs.

In our stochastic folding pathway model, which we call the *distinct-arc model*, we assume that the $k$ initially-present arcs are between bases $A_{1}\dots A_{k}$ and bases $\bar{A}_{k}\dots \bar{A}_{1}$ (in reverse order to avoid pseudoknots), and the only arcs that can be added or removed are arcs between $A_i$ and $\bar{A}_{i}$. We define the stochastic model by assigning a propensity $\alpha$ to adding an arc, and a propensity $1/\alpha$ to removing an arc, for some constant $\alpha > 1$. Then at a step of the pathway, if the current structure has $k-i$ arcs, the probability of adding an arc is $i\alpha / (i\alpha + (k-i)/\alpha ) = i\alpha ^2 / (i\alpha ^2 + k-i)$, with each missing arc being equally likely to be added, and probability of removing an arc is $(k-i)/(i\alpha ^2 + k-i)$, with each of the $i$ currently present arcs being equally likely to be removed.

In measuring the time to follow a pathway, we simply count the number of arc addition and removal steps, since for our simple model, the expected time to follow a step is constant (depending on $\alpha$).

#### Claim 13

Let $$ B ( k )$$ be the expected time to ascend a barrier of size $$ k $$ in the distinct-arc model. Then

1 $$\begin{aligned} 2(\alpha ^{2} + 1)^{k - 1} - 2 + k\le B(k) \le 2k(\alpha ^{2} + 1)^{k - 1} - k. \end{aligned}$$

#### Proof

We will construct a Markov chain with $k + 1$ states $0,\dots ,k$, where state $i$ represents the configuration with $i$ of the $k$ arcs removed. Assume that we are at state $i$. Then the probability that one of these $i$ arcs will be added back is $p_{i,i-1} = \frac{i\alpha ^{2} }{i\alpha ^{2} + k - i }$ and the probability that any of the remaining $k - i$ arcs will be removed is $p_{i,i+1} = \frac{k - i}{i\alpha ^{2} + k - i }$. Let $s_{i} = p_{i,i-1}/ p_{i,i+1} = \frac{i\alpha ^{2}}{k - i}$. Note that $1/p_{i,i+1} = \frac{i\alpha ^{2} + k - i}{k - i} = \frac{i\alpha ^{2}}{k - i} + 1 = s_{i} + 1$.

We would like to calculate the expected time until the Markov chain visits state $k$. We will use the following result:

#### Theorem 4

[Theorem 1.3.5 in Norris (1997)] *Let* $A$ *be a set of states. Let* $T_{i}^{A}$ *be the expected time to hit (visit) a state in* $A$ *when starting the Markov chain from state* $i$. *Then the vector of mean hitting times* $(T_{i}^{A})_{i = 0}^{k}$ *is the minimal non-negative solution to the system of linear equations*

$$\begin{aligned} \begin{aligned} T_{i}^{A}&= 0\qquad&\text {for }i\in A\\ T_{i}^{A}&= 1 + \sum _{j}p_{ij}T_{j}^{A}\qquad&\text {for }i\notin A \end{aligned} \end{aligned}$$

In our case, we set $A = \{k\}$ and we are interested the value of $T_{0}^{A}$. Let $T_{i} := T_{i}^{\{k\}}$. We have the following system of equations:

2 $$\begin{aligned} \begin{aligned} T_{0}&= 1 + p_{0,1}T_{1}\\ T_{i}&= 1 + p_{i,i-1}T_{i - 1} + p_{i,i+1}T_{i + 1} \quad&\text {for }i = 1,\dots ,k - 1\\ T_{k}&= 0 \end{aligned} \end{aligned}$$

Note that dividing both sides of (2) for $i = 1,\dots ,k - 1$ by $p_{i,i+1}$, we get

3 $$\begin{aligned} (s_{i} + 1)T_{i} = s_{i} + 1 + s_{i}T_{i - 1} + T_{i + 1}. \end{aligned}$$

Let $d_{i} = T_{i - 1} - T_{i} - 1$. Then by (3), for any $i = 1,\dots ,k - 1$,

4 $$\begin{aligned} d_{i + 1} = T_{i} - T_{i + 1} - 1 = s_{i}(T_{i - 1} - T_{i} + 1) = s_{i}(d_{i} + 2). \end{aligned}$$

By (2), $d_{1} = T_{0} - T_{1} - 1 = 0$, since $p_{0,1} = 1$. Let $(n)_{i} = n(n - 1)\dots (n - i + 1)$. It is easy to verify by induction that

5 $$\begin{aligned} d_{j} = 2 \sum _{i = 1}^{j - 1} \frac{(j - 1)_{i}}{(k - j + i)_{i}}\alpha ^{2i} \end{aligned}$$

It follows by the binomial theorem and the fact that $$\frac{(k - 1)_{i}}{(i)_{i}} = \left( {\begin{array}{c}k - 1\\ i\end{array}}\right) $$ that $d_{k} = 2(\alpha ^{2} + 1)^{k - 1} - 2$. Unfortunately, it is not possible to calculate exactly the remaining $d_{j}$’s, however, we will show that for any $j$, $d_{j}\le d_{k}$.

Note that $0 = s_{0}< s_{1}< \dots < s_{k - 1} = (k - 1)\alpha ^{2}$, and let $p$ be the integer such that $s_{p - 1}\le 1 < s_{p}$. Then for any $j\ge p$, $d_{j + 1} = s_{j}(d_{j} + 2)\ge d_{j}$, hence, $d_{j}\le d_{k}$. Next we will show by induction on $j$ that for any $j < p$, $d_{j}\le 2(j - 1)$. Clearly, this is true for $j = 0$. Assume that $j < p$ and $d_{j - 1}\le 2(j - 2)$. Then $d_{j} = s_{j - 1}(d_{j - 1} + 2)\le d_{j - 1} + 2\le 2(j - 2) + 2 = 2(j - 1)$. The claims follows, and hence, for any $j < p$, we have $d_{j}\le 2j - 2\le 2(k - 1)\le (\alpha ^{2} + 1)^{k - 1} - 1 = d_{k}$ (since $\alpha ^{2} + 1 > 2$).

Note that

6 $$\begin{aligned} \sum _{j = 1}^{k}d_{j} = \sum _{j = 1}^{k} (T_{j - 1} - T_{j} - 1) = T_{0} - T_{k} - k = T_{0} - k. \end{aligned}$$

Finally, we can bound the expected time until all arcs are removed ($T_{0}$) as follows. Since $d_{k}\le \sum _{j = 1}^{k} d_{j}\le kd_{k}$, we have

7 $$\begin{aligned} 2(\alpha ^{2} + 1)^{k - 1} - 2 + k\le T_{0}\le 2k(\alpha ^{2} + 1)^{k - 1} - k. \end{aligned}$$

Since $T_0 = B(k)$, the expected time to ascend a barrier of size $k$ in the distinct-arc model, the result follows. $\square$

**Fig. 4:**
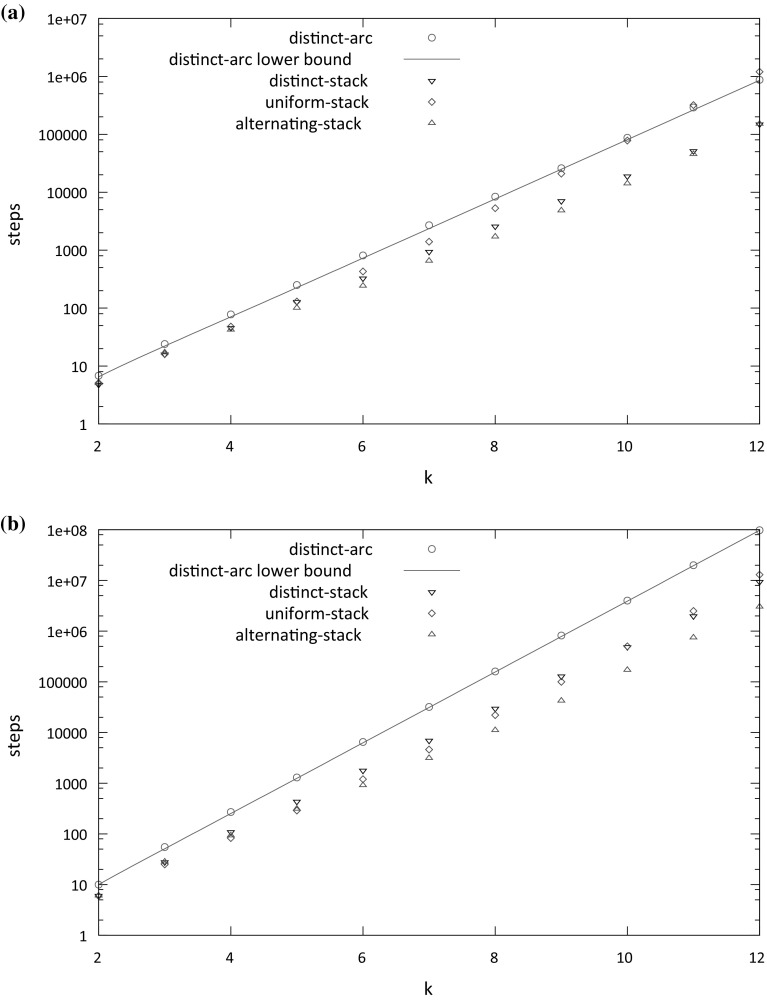
Dependency of the average number of steps to scale a barrier on the size $k$ of the barrier for the distinct-arc, distinct-stack and uniform-stack models when the propensity rate is **a** $\alpha = 1.5$ and **b** $\alpha = 2$. The average is taken over 1000 experiments

**Fig. 5:**
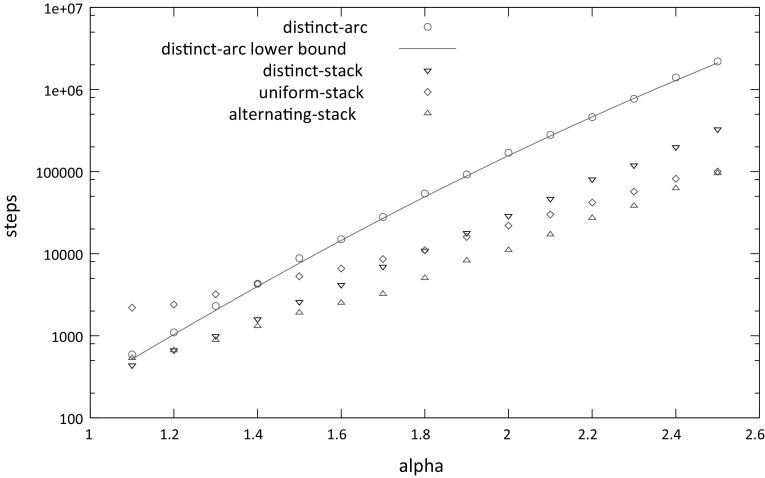
Dependency of the average number of steps to scale a barrier of size $k=8$ on the propensity rate $\alpha$, for the distinct-arc, distinct-stack and uniform-stack models. The average is taken over 1000 experiments

### Simulations of other energy models

It is more challenging to prove bounds on the expected time to scale a barrier in stacked pair energy models, so we instead provide some insights using simulations. We consider the following models:

- *Distinct-stack model* Like the chain model, bases $A_{1}\dots A_{k}$ are initially paired with $\bar{A}_{k}\dots \bar{A}_{1}$ (in reverse order), and the only arcs that can be added or removed are arcs between $A_i$ and $\bar{A}_{i}$. In constrast with the distinct-arc model, the propensity of adding an arc is $\alpha ^{2}$ if the arc forms two new stacked pairs, $\alpha$ if the arc forms one new stacked pair with another arc, and is 1 otherwise, while the propensity of removing an arc is $1/\alpha ^{2}$ if the arc forms two stacked pairs, $1/\alpha$ if the arc forms a stacked pair with another arc and is 1 otherwise, where again $\alpha > 1$.
- *Uniform-stack model* This model is similar to the distinct-stacked model, except that for any $1 \le i,j \le k$, $A_i$ can pair with any $\bar{A}_j$ as long as no two arcs cross. Equivalently, all of the $A_i$’s are the same base and all of the $\bar{A}_j$’s are the complement of the $A_i$’s.
- *Alternating-stack model* This model is similar to the previous stacked models, except that for any $1 \le i,j \le k$, $A_i$ can pair with any $\bar{A}_j$ as long as $i$ and $j$ have the same parity and no two arcs cross. Equivalently, all $A_i$’s in odd (even) positions are the same base and each $\bar{A}_{i}$ is the complement of $A_{i}$.

Figure 4 shows how the average number of steps to scale a barrier of size $k$ depends on $k$ for each model, where propensity rates are $\alpha = 1.5$ and $\alpha = 2$. For both values of $\alpha$, the average number of steps needed by the distinct-stack model is less than than the average for the distinct-arc model. This may be because in the distinct-arc model, any removed arc has propensity $\alpha$ to be added, while in the distinct-stack model, adding arcs that do not extend one of the the current stacks of arcs has propensity 1, so the overall probability of adding an arc is smaller in the stack model (especially when only few arcs remain).

For $\alpha = 1.5$, the number of steps needed by uniform-stack model starts to surpass the number needed by the distinct-arc model for $k=11$; however for $\alpha =2$ it tracks closely with the lower number needed by the distinct-stack model. While it is difficult to be sure based on the plots, it seems plausible that the time to scale a barrier of size $k$ grows exponentially with $k$ for all three models.

Figure 5 shows how the average number of steps needed to scale a barrier of size $k=8$ depends on the propensity rate $\alpha$. Not surprisingly, the average grows as $\alpha$ increases. However, the rate of increase is significantly less for the uniform-stack model than for the distinct-arc and distinct-stacked models. This may be because when arcs are added in the uniform-stack model, they may prevent other arcs from being added; for example, if $A_1$ is paired with $\bar{A}_k$ then any additional arcs would form a pseudoknot. Impediments to the addition of arcs would make it easier to scale the barrier.

### Time needed to follow a low-barrier pathway

In the rest of this section, suppose that the constants $C$ and $\epsilon$ of Corollary 6 are fixed. Let $\mathbb {E}[\mathrm {low}]$ be the expected time to reach the target structure from the initial structure of the switch and lock sequence while following a low-barrier pathway. In the stochastic setting, it does not make sense to ask when is the target structure reached exactly, i.e., when are all of the arcs of the target structure—and no other arcs—present. Instead, by “reaching the target structure”, we mean that all of the bands of the initial lock structure are unlocked, i.e., no center arcs are present in the lock structure. By “following a low-barrier pathway” we mean following a pathway whose barrier is at most the bound $(2 -\epsilon )C \log n - O(1)$ of Corollary 6.

We will first show, using informal arguments, that $\mathbb {E}[\mathrm {low}]$ is in $O(n \log ^2 n \gamma ^k)$ for some $\gamma >1$ (where $k = C\log n$). We then apply Markov’s inequality to argue that with probability $1-1/\varTheta (n)$, the target is reached while following a low-barrier pathway within $f(n) = n^2 \log ^2 n \gamma ^k$ time.

To get from the initial to the target structure requires the removal (unlocking) of all $B$ lock bands of the initial structure. Recall from Claim 7 that the bands must be removed from the outside in, in order to keep the barrier low. Because the folding process is stochastic, once $i$ bands are removed, the pathway could regress by adding a band back in, or the pathway could progress by removing the $(i+1)$-st band (if the pathway regresses, the band added back in may not necessarily be the most recently removed band, or even a center band, but this detail is not significant in our argument).

#### Removal or addition of a single band

Within a band removal or addition phase of a low-barrier pathway, a reconfiguration of the switch may be necessary; it will certainly be necessary at some point between the first removal of band $i$ and the first removal of band $i+1$. Recall that reconfiguring the switch involves three subphases, as described in Claim 4: a barrier-$k$ ascent, followed by branch migration and a barrier-$k$ descent. We argue that the overall expected time for switch reconfiguration is dominated by the barrier-$k$ ascent. To estimate the expected time for each subphase, we first estimate the number of “progression-specific” steps needed for switch reconfiguration, i.e., the arc addition and removal steps that either make progress, or undo progress, in the subphases of reconfiguring the switch. The branch migration process, resembling a random walk, is expected to take $O(n^2)$ progression-specific steps, and barrier descent takes $\varTheta (n)$ steps. These are both negligible compared with the expected time for the barrier-$k$ ascent, which we estimate as being $\varTheta (\gamma ^k)$ for some $\gamma > 1$, based on the results of Sects. 4.1 and 4.2. In addition to the progression-specific steps described so far, other arcs may form and break that do not affect the progress of switch reconfiguration, e.g., in the lock. Since there are $\varTheta (n)$ arcs in any structure along a low-barrier pathway, and any of these may break, the slow-down they introduce to switch progression is bounded by a factor of $\varTheta (n)$. Thus, overall, the expected time for switch reconfiguration should be $O(n \gamma ^k)$.

In addition to the switch reconfiguration subphase, band removal will also involve lock-specific subphases, namely two barrier-$k$ ascents, two barrier-$k$ descents, and a branch migration process as described in Claim 4. Band addition, when the pathway regresses, involves similar phases. Again, the expected time for these subphases is dominated by the barrier-$k$ ascents. Thus overall, addition or removal of a single band is expected to complete in $O(n \gamma ^k)$ steps.

#### Unlocking all of the initial lock bands

Recall that the bands must be removed from the outside in, in order to keep the barrier low, and that the time for band removal or addition is dominated by the time to do barrier-$$ k $$ ascents. Once $$ i $$ bands of the initial lock structure are unlocked, it is roughly equally likely that the process will regress, by adding a band, or that the process will progress by reconfiguring the switch. Similarly, if the switch is reconfigured, it’s roughly equally likely that the process will regress, by “un”-reconfiguring the switch, or that the process will progress by removing another lock band. Thus a reasonable approximation of the folding process is that it is an unbiased random walk of barrier-$$ k $$ ascents that ends when all $$B$$ bands are removed. Since $$ O (1)$$ ascents are needed per band removal, the expected number of phases in such a random walk is $$\varTheta (B^2)$$. Thus, the overall expected time $$\mathbb {E}[\mathrm {low}]$$ to follow a low-barrier pathway and reach the target is in $$O(B^2 n \gamma ^k) = O(n \log ^2 n \gamma ^k)$$.

#### Applying Markov’s inequality

Let $$ p ( n )$$ be any function whose range is [0,1]. Then with probability at least $$1-p(n)$$, the time to reach the target, while following a low-barrier pathway, is at most $$\mathbb {E}[\mathrm {low}]/p(n)$$. This follows directly from Markov’s inequality, which states that if $$ X $$ is a nonnegative random variable then

$$\begin{aligned} \mathbb {P}(X \ge a) \le \frac{\mathbb {E}(X)}{a}. \end{aligned}$$

We simply choose $X$ to be the time to reach the target while following a low-barrier pathway, and choose $a$ to be $\mathbb {E}[\mathrm {low}]/p(n)$.

Thus, with probability $1-1/\varTheta (n)$, the target is reached while following a low-barrier pathway within $f(n) = n^2 \log ^2 n \gamma ^k$ time.

### On the likelihood of following a low-barrier pathway

Here we argue that a low-barrier pathway from the initial to target structure, which must have $$\varOmega (n \log n)$$ distinct secondary structures due to switch reconfigurations and trans-arcs, is more likely to be followed than a pathway that removes even one lock of the band via a barrier $$2 k $$-ascent.

Let $$p_{2k}(n)$$ be the probability of ascending a $$2 k $$-barrier within $$f(n) = n^2 \log ^2 n \gamma ^k$$ time, where $$k = \varTheta (\log n)$$. We can bound the expected time for a $$2 k $$-barrier ascent as a function of $$p_{2k}(n)$$ and $$ f ( n )$$ as follows. If, after $$ f ( n )$$ steps of a pathway the ascent has not completed, the probability that it will complete in the next $$ f ( n )$$ steps is at least $$p_{2k}(n)$$, because the worst case is that after $$ f ( n )$$ steps the current structure has all $$2 k $$ base pairs. More generally, if the ascent has not completed after $$ if ( n )$$ steps, then the probability that it is completed in the next $$ f ( n )$$ steps is at least $$p_{2k}(n)$$. Therefore the expected number of steps to complete the ascent is at most

$$\begin{aligned} \sum _{i=0}^{\infty } \textit{if}\,(n) p_{2k}(n) (1-p_{2k}(n))^{i-1} = f(n)/p_{2k}(n). \end{aligned}$$

From Sect. 4.1, the expected time to ascend a barrier of size $$2 k $$ is at least $$\gamma ^{2k}$$ (using the same $$\gamma $$ as in Sect. 4.3). Thus $$\gamma ^{2k} < f(n) /p_{2k}(n)$$. Therefore,

$$\begin{aligned} p_{2k}(n) < f(n)/\gamma ^{2k} = n^2 \log ^2 n / \gamma ^{k} = 1/n^{\varTheta (1)}, \end{aligned}$$

since $k = C\log n$ and we can choose constant $C$ to be larger than $6/\log \gamma$.

Thus for sufficiently large $$ n $$, the probability of ascending a $$2 k $$-barrier within $$ f ( n ) = n^2 \log ^2 n \gamma ^k$$ time is less than $$1/n^{\varTheta (1)}$$, while the probability that a low-barrier pathway will be followed within $$ f ( n )$$ time is at least $$1-1/\varTheta (n)$$.

To summarize, we have argued that reaching the target structure from the initial structure is significantly more likely via a low-barrier pathway with switch reconfigurations, than by direct removal of even a single band of the lock. However, the arguments of this section do not address the possibility of pathways from initial to target structure that avoid both $$\varOmega (n \log n)$$ switch reconfigurations and a barrier-$$2 k $$ ascent. Corollary 6 does rule out any such pathway with barrier less than $$(2-\epsilon )k - O(1)$$, and the arguments of this section apply equally well to show that a low-barrier pathway is significantly more likely than a barrier $$(2-\epsilon )k - O(1)$$-ascent. But it would be interesting to also show that pathways from initial to target that avoid switch configurations not only have a higher barrier, but more specifically must ascend a barrier of size at least $$(2-\epsilon )k - O(1)$$.

## Design challenges

Key properties of our design rest on simplifying assumptions of our model: our energy model ignores loop penalties, pseudoknots, and intra-molecular bonds. Because state-of-the-art nucleic acid energy models have thousands of parameters, it would be very difficult to reason mathematically about properties of our design with respect to such models. While our simplifying assumptions made it feasible for us to develop a rigorous proof, they may also result in design weaknesses that cause problems in a real experimental setting. Here we consider how some such weaknesses might be addressed.

### Pseudoknots

With our current design, it’s possible that bands of the lock from both the initial and final structures could bind simultaneously, thereby forming pseudoknots. Then, our initial and target structures would not be minimum free energy structures. Moreover, the switch could be rendered ineffective if the innermost unpaired bases could stably pair with the outermost complementary bases of the switch. (I.e., in Fig. 1a, the unpaired ${\mathsf {U}}$’s of the switch could pair with the unpaired ${\mathsf {A}}$’s of the switch.) To avoid these problems, the design could be adapted so that some band regions of the lock and switch have their own internal structure, making pseudoknot formation thermodynamically unfavourable. For example, a hairpin structure within the ${\mathsf {X}}$ region of a band could be more stable than a pseudoknotted structure involving both ${\mathsf {C}}\cdot {\mathsf {G}}$ and ${\mathsf {X}}\cdot {\mathsf {Y}}$ bands, yet less stable than a pseudoknot-free band between the ${\mathsf {X}}$ and ${\mathsf {Y}}$ regions once the intervening ${\mathsf {C}}\cdot {\mathsf {G}}$ bands are removed.

### Inter-molecular bonds

If bonds form between multiple copies of the design, the lock of one molecule $M$ could be unlocked via a short pathway by forming base pairs with switches of two additional molecules $M'$ and $M''$. This would be possible, even while avoiding pseudoknotted structures, if the switch of $M'$ is fixed in the left position (as in Fig. 1a) while the switch of $M''$ is fixed in the right position (as in Fig. 1b). The switches would not need to repeatedly change from left to right position and thus the superlinear pathway of our design would be lost. This problem could be avoided in an experimental setting, if individual copies of the molecule are isolated from each other, for example by tethering them to a surface or running the experiment with a very low concentration of our molecules.

### Energy model

How might one adapt our design (or other future designs of complex folding pathways) to work with more realistic energy models? The sequence design could be iteratively tested and modified via folding pathway simulators such as Multistrand (Schaeffer et al. 2015) or RNAtabupath (Dotu et al. 2010), but it could be prohibitively time consuming to find robust solutions to design flaws with such an approach. Instead, a multi-level design and testing approach could first simulate pathways at a domain level, where complementary regions are represented by abstract symbols over a large alphabet (such as our 8-letter alphabet). Tests at the domain level would check for unexpected interactions between complementary domains that are design flaws, e.g., short-cuts to our designed pathway. Such tests would be akin to the DSD simulator for DNA strand displacement systems (Lakin et al. 2011), but for more sophisticated pathways than strand displacement. At a lower level of detail, abstract domains of a working design could then be mapped to nucleic acid sequences so that there is a low probability of binding between a domain $d$ and another domain $d'$ that is not complementary to $d$, or between $d$ and the concatenation of two non-complementary domains $d'$ and $d''$.

Yet another issue that is not addressed in our methods is how to initially arrange for the molecule to form the initial structure, before the folding rearrangement can proceed. One way to do this could be to initially add complements to the $\mathsf {X}$ and $\mathsf {Q}$ domain sequences, so that these are paired with their complements. If these sequences are bound, the only minimum free energy structure for the rest of the lock sequence is the initial structure. A toehold-mediated strand displacement mechanism could then remove the complementary strands, whereupon the planned folding pathway would be followed. The pathway reaches the target structure only if the innermost bases of the lock sequence are exposed, i.e., are no longer inside a loop. By inserting a distinct sequence at the center of the lock, it should be possible to detect, i.e., read out, when the target is reached.

Another drawback of our design is that the target configuration is just one of many stable (MFE) structures, and so the molecule’s pathway may rarely visit the target. This issue could be addressed by adding additional bases to the sequence that can form base pairs only when the target is reached, thereby making the target more stable. For example, bases could be added at the very center and outermost regions of lock that can only pair with each other once all of the ${\mathsf {C}}\cdot {\mathsf {G}}$ and ${\mathsf {A}}\cdot {\mathsf {U}}$ bands are removed. This adaptation of the design could be generalized so that, as successive ${\mathsf {C}}\cdot {\mathsf {G}}$ or ${\mathsf {A}}\cdot {\mathsf {U}}$ bands are removed and replaced by ${\mathsf {X}}\cdot {\mathsf {Y}}$ and ${\mathsf {P}}\cdot {\mathsf {Q}}$ bands, the structure becomes increasingly stable and thus the folding pathway is energetically biased towards the target structure.

## Conclusions

In this work we have presented the design of RNA molecules whose folding pathways from a given initial to target structure are expected to visit a number of distinct structures that grows superlinearly in the strand length. Our design is based on a conceptually simple lock and switch mechanism, and advances current understanding of properties of indirect folding pathways (Dotu et al. 2010; Morgan and Higgs 1998).

The energy barrier of the desired long pathway grows (logarithmically) with the length of the molecule, assuming that it is also necessary to ensure that the barrier gap between the desired pathways and alternative shorter pathways also grows with the length of the molecule. It would be interesting to find a design in which the energy barrier of the desired low-barrier pathway is independent of the length of the molecule, while still achieving a barrier gap that grows with the length of the molecule.

Yet a different way improvement would be to obtain a design in which the length of the desired low-barrier folding pathway grows exponentially with the length $n$ of the strand, rather than proportional to $n \log n$.

Our design suggests that it may be possible to do non-trivial volume-efficient computations with single-stranded nucleic acids. To see how a volume-efficient counter could be useful for this purpose, it is helpful to recall our earlier work (Condon et al. 2012), where we designed a multi-stranded, volume-efficient counter using strand displacement primitives. We were then able to incorporate that design into a general method for volume-efficient simulation of space bounded computations (Thachuk and Condon 2012). For example, to test the truth of a Boolean formula, the role of the binary counter is to enumerate all possible truth assignments of the variables of the formula. A single-stranded counter would similarly be useful in enumerating states that should be explored as part of another computation. Alternatively, a counter might be useful to coordinate proper order of computational steps, for example, to ensure that certain reactions only happen after enough time has passed for preparatory steps. As noted above, the design could be modified so that a unique subsequence at the center of the lock sequence is exposed only when the target structure is reached. This subsequence could then react to trigger further reactions.

A different direction for further research would be to develop a higher level language for design of single-strand RNA molecules as the concatenation of abstract domains, i.e., substrands represented by a single symbol (just as DSDs are often described as domains) and a “compiler” that could translate a sequence of domain symbols into an RNA strand by associating a string over $\{\mathsf {A,C,G,U}\}$ with each domain symbol. The compiler would need to do this translation while preserving folding pathway properties of the abstract design. Such a compiler could help avoid the cumbersome work of proving correctness at the sequence level, and possibly ease the task of doing empirical studies. Ultimately, the goal of tools that provide useful layers of abstraction would be to facilitate 4D-RNA printing—the design of RNA strands that not only have desired secondary and even 3D structures, but in fact change their 3D shapes in desired ways over time.

## Appendix

Here, we give proofs of claims that are auxiliary to the main thread of argument and to claims that were stated without proof in the text. Claims with numbers in parentheses are mentioned in the main text. Claims with numbers that are not in parentheses are claims that were not mentioned in the main text. In several proofs we will only focus on the left–left case; the right–right case is symmetric: consider a structure with right–right trans-arcs, exchange the order the switch and lock and reverse the order of the whole sequence. It is easy to check that we have the left–left case of a sequence with permuted bases.

## The 8-letter alphabet design

### Bounding the arc count in structures with trans-arcs

#### Switch

##### Claim (1)

All MFE structures of ${\text {switch}}(k,A)$ have ${\text {MAC}}{}_{\mathrm {switch}}(k,A){}:= (A- 1)k$ arcs.

##### Proof

If no base of region $L_1$ is paired then the total number of arcs is at most $(A- 1)k$. Otherwise, assume there is an arc from region $L_1$ to region $R_\sigma$, where $\sigma$ is even. It follows that bases in region $R_1$ are unpaired, hence again the total number of arcs is at most $(A- 1)k$. Since the initial and target structures of the switch demonstrate that structures with $(A- 1)k$ arcs exist and achieve this count, then we know that the MFE structures have these many arcs. $\square$

##### Claim 14

The number of intra-switch arcs in any structure $S$ of ${\text {switch-lock}}(k,A, B)$ is at most ${\text {MAC}}{}_{\mathrm {switch}}(k,A){}$ if $c= 1$ and is at most ${\text {MAC}}{}_{\mathrm {switch}}(k,A){}+ (2 - c)k - i$ if $c> 1$.

##### Proof

There are no intra-switch arcs involving a base to the left of $L_{c,i}$. If $c= 1$, by Claim 1, the switch can form the maximal number of intra-switch arcs, namely ${\text {MAC}}{}_{\mathrm {switch}}(k,A){}$. If $c> 1$, the number of intra-switch arcs to the right of $L_{c,i}$ is at most the number of $\mathsf {U}$’s and $\mathsf {G}$’s to the right of $L_{c,i}$ in the switch which is $(A- c)k + (k - i) = {\text {MAC}}{}_{\mathrm {switch}}(k,A){}+ (2 - c)k - i$. $\square$

##### Claim 15

Consider any structure of ${\text {switch-lock}}(k,A, B)$. Then the number of arcs involving bases of the switch is at most ${\text {MAC}}{}_{\mathrm {switch}}(k,A){}+ t\le {\text {MAC}}{}_{\mathrm {switch}}(k,A){}+ k$.

##### Proof

Note that, by Claim 1, ${\text {MAC}}{}_{\mathrm {switch}}(k,A){}=(A-1)k$. If there are no trans-arcs, then $t= 0$ and the number of arcs involving bases of the switch is at most ${\text {MAC}}{}_{\mathrm {switch}}(k,A){}$, and so the claim holds. If there are trans-arcs, either all are left–left or right–right.

If $$ c= 1$$ then $$T= t\le k$$ and the claim follows easily. Suppose that $$c> 1 $$. The number of bases of regions $$L_{2},\dots ,L_{c}$$ that are involved in trans-arcs is $$T- t$$. Note that bases of region $$L_1$$ cannot form intra-switch arcs. The number of $$\mathsf {G}$$’s and $$\mathsf {U}$$’s in the regions $$L_{2},\dots ,L_{A}$$ that are not involved in trans-arcs is $$(A- 1)k - (T- t)$$, and so the number of intra-switch arcs is at most $$ {\text {MAC}}{}_{\mathrm {switch}}(k,A){}- T+ t$$. Thus the total number of arcs that involve bases of the switch is at most $${\text {MAC}}{}_{\mathrm {switch}}(k,A){}- T+ t+ T= {\text {MAC}}{}_{\mathrm {switch}}(k,A){}+t$$.

The proof when all trans-arcs are right–right follows by symmetry. $\square$

#### Lock

##### Claim (2)

All MFE structures of ${\text {lock}}(k, B)$ have ${\text {MAC}}{}_{\mathrm {lock}}(k,B){}:= 2k B$ arcs.

##### Proof

Consider an MFE structure $S$ of the lock. Let $\sigma$ ($\sigma '$) be the largest index such that no base in region $l_{\sigma }$ (region $r_{\sigma ' }$) is forming an arc. Clearly, for any $d > \sigma$, there are no $y_{d}$-arcs and for any $d > \sigma '$, there are no $p_{d}$-arcs. Hence, $S$ has at most $k\sigma$ $\mathsf {Y}$-arcs and at most $k\sigma '$ $\mathsf {P}$-arcs. On the other hand, we have $2k(B- \sigma )$ bases in $l$-regions and $2k(B- \sigma ' )$ bases in $r$-regions which can form arcs. Hence, the number of center arcs is at most $2k\min (B- \sigma ,B- \sigma ' ) = 2k(B- \max (\sigma ,\sigma ' ))$. The total number of arcs in $S$ is at most

8 $$\begin{aligned}&k\sigma + k \sigma ' + 2k(B- \max (\sigma ,\sigma ' )) \le k \sigma + k \sigma '\nonumber \\&\quad + 2k\left( B- \tfrac{k \sigma + k\sigma ' }{2k}\right) = 2kB. \end{aligned}$$

Since the initial and target structures of the lock achieve this arc count, all MFE structures have this many arcs. $\square$

##### Claim 16

The number of intra-lock arcs in any structure $S$ of ${\text {switch-lock}}(k,A, B)$ with trans-arcs is at most ${\text {MAC}}{}_{\mathrm {lock}}(k,B){}- qk + \max \{k-j,0\}$.

##### Proof

We count the intra-lock arcs in the left–left case (the right–right case follows by symmetry) as follows.

- $\mathsf {X}\cdot \mathsf {Y}\,arcs$ These are only possible to the left of the trans-arcs. There are at most $(b{-}q)k$ such arcs.
- $\mathsf {P}\cdot \mathsf {Q}{-}arcs$ There are at most $ak$ such arcs.
- $Center{-}arcs$ If $a<b$, the number of center arcs is bounded by the number of available bases in $l$-regions, which is $(B- b)2k + (2k - j)$. If $a\ge b$, the number of center-arcs is bounded by the number of available bases in $r$-regions, which is $(B- a)2k$.

Summing all three types of intra-lock arcs, if $a<b$, the total is

$$\begin{aligned} (b- q)k + ak + (B- b)2k + (2k-j) \le B2k - qk + k-j\end{aligned}$$

(the inequality is obtained by setting $a= b-1$, since this maximizes the quantity for all $a < b$). If $a\ge b$ the total is

$$\begin{aligned} (b- q)k + ak + (B- a)2k \le B2k - qk \end{aligned}$$

(the inequality is obtained by setting $a= b$).

The total number of intra-lock arcs is therefore at most

$$\begin{aligned} B2k - qk + \max \{k-j,0\}= & {} {\text {MAC}}{}_{\mathrm {lock}}(k,B){}- qk \\&+ \max \{k-j,0\}, \end{aligned}$$

reflecting the fact that the best choice of $a$ (as being $b-1$ or $b$) depends on whether $j\le k$. $\square$

#### Switch and Lock

##### Claim (3)

All MFE structures of the switch and lock sequence ${\text {switch-lock}}(k,A, B)$ have ${\text {MAC}}{}(k,A, B){}:= {\text {MAC}}{}_{\mathrm {switch}}(k,A){}+ {\text {MAC}}{}_{\mathrm {lock}}(k,B){}$ arcs. The initial and target structures are MFE structures.

##### Proof

Consider a structure $S$ of the switch and lock. If $S$ does not contain any trans-arcs, then the claim easily follows by Claims 1 and 2. By Claims 15 and 16, we have

$$\begin{aligned} {\text {AC}}(S)\le & {} {\text {MAC}}{}_{\mathrm {switch}}(k,A){}+ t+ {\text {MAC}}{}_{\mathrm {lock}}(k,B){}- qk\\&+ \max \{k - j,0\}. \end{aligned}$$

If $$q\ge 2$$, since $$t\le k$$ and $$k - j< k$$, we have $${\text {AC}}(S)\le {\text {MAC}}{}_{\mathrm {switch}}(k,A){}+ {\text {MAC}}{}_{\mathrm {lock}}(k,B){}$$. If $$ q= 1$$, since $$t\le j$$ and $$t\le k$$, we have

$$\begin{aligned} {\text {AC}}(S)&\le {\text {MAC}}{}_{\mathrm {switch}}(k,A){}+ {\text {MAC}}{}_{\mathrm {lock}}(k,B){}\\&\quad + \max \{t- j,t- k\} \\&\le {\text {MAC}}{}_{\mathrm {switch}}(k,A){}+ {\text {MAC}}{}_{\mathrm {lock}}(k,B){}. \end{aligned}$$

Since the initial and target structures achieve this bound, they are MFE structures. $\square$

##### Claim (5)

Consider a structure $S$ for ${\text {switch-lock}}(k,A, B)$, where $S$ has trans-arcs. Suppose that $c+ q> 4$. Then ${\text {AC}}(S)\le {\text {MAC}}{}(k,A, B){}- 2k$.

##### Proof

We prove the claim for the case where $S$ has left–left trans-arcs; the right–right trans-arcs case is symmetric. We consider several cases for the values of $c$ and $q$, calculate the maximum possible number of trans-arcs for each case, and then add this maximum to the bound on intra-switch arcs from Claim 14 and the bound on intra-lock arcs from Claim 16 to bound the total number of arcs as follows:

9 $$\begin{aligned}&{\text {MAC}}{}_{\mathrm {switch}}(k,A){}+ (2-c)k -i+ {\text {MAC}}{}_{\mathrm {lock}}(k,B){}- qk\nonumber \\&\qquad + \max \{k-j,0\} + T\nonumber \\&\quad = {\text {MAC}}{}(k,A, B){}- (c+ q- 2)k -i\nonumber \\&\qquad + \max \{k,j\} -j+ T. \end{aligned}$$

To reduce the number of cases, we observe that if the leftmost region of the lock that is involved in trans-arcs contains $\mathsf {A}$’s, then $c$ must be even, since the rightmost bases of the switch that pair with the lock must be $\mathsf {U}$’s and $\mathsf {U}$’s are in even-numbered regions of the switch. Similarly, if the leftmost region of the lock that is involved in trans-arcs contains $\mathsf {C}$’s, then $c$ must be odd. For example, if $q= 2$ and $c= 4$, it must be that the two regions of the lock that are involved in trans-arcs contain $\mathsf {A}$’s followed by $\mathsf {C}$’s. To improve readability we will use ${\text {MAC}}{}$ instead of ${\text {MAC}}{}(k,A, B){}$ in the formulas.

- $q= 1$ and $c= 4$ The number of trans-arcs is at most $j\le k+i$; this bound is attained when the single lock region that is involved in trans-arcs contains $\mathsf {A}$’s, which are paired with $k$ $\mathsf {U}$’s from the second region of the switch and $i$ $\mathsf {U}$’s from the fourth region. By expression 9, the total number of arcs is at most
  $$\begin{aligned} {\text {MAC}}{}- 3k -i+ \max \{k,k+\,i\} = {\text {MAC}}{}- 2k. \end{aligned}$$
- $q= 1$ and $c\ge 5$ The number of trans-arcs is at most $j\le 2k$. By expression 9, the total number of arcs is at most
  $$\begin{aligned} {\text {MAC}}{}- 4k -i+ \max \{k,2k\} \le {\text {MAC}}{}- 2k. \end{aligned}$$
- $q= 2$ and $c= 3$ The number of trans-arcs is at most $j+i$, where $j\le k$; this bound is attained when the two regions of the lock that are involved in trans-arcs contain $\mathsf {C}$’s followed by $\mathsf {A}$’s, with the $\mathsf {C}$’s of the lock paired with $i$ $\mathsf {G}$’s in region 3 of the switch and the $j$ $\mathsf {A}$’s of the lock paired with the at most $k$ $\mathsf {U}$’s in region 2 of the switch. By expression 9, the total number of arcs is at most
  $$\begin{aligned}&{\text {MAC}}{}- 3k -i+ \max \{k,j\} + i= {\text {MAC}}{}- 3k -i\\&\quad +\,k + \,i= {\text {MAC}}{}- 2k. \end{aligned}$$
- $q= 2$, $c= 4$ and $j\le k$ The number of trans-arcs is at most $i+j+k$; this bound is attained when the two regions of the lock that are involved in trans-arcs contain $\mathsf {A}$’s followed by $\mathsf {C}$’s, with the $j$ $\mathsf {C}$’s of the lock paired with $\mathsf {G}$’s in region 1 of the switch and the $\mathsf {A}$’s of the lock paired with the at most $k+i$ $\mathsf {U}$’s in regions 2 and 4 of the switch. By expression 9, the total number of arcs is at most
  $$\begin{aligned}&{\text {MAC}}{}- 4k -i+ \max \{k,j\} + i\\&\quad +\, k = {\text {MAC}}{}- 4k + k + k = {\text {MAC}}{}- 2k. \end{aligned}$$
- $q= 2$, $c= 4$ and $j> k$ The number of trans-arcs is at most $i+j$; this bound is attained when the two regions of the lock that are involved in trans-arcs contain $\mathsf {A}$’s followed by $\mathsf {C}$’s, with the $j$ $\mathsf {C}$’s of the lock paired with $\mathsf {G}$’s in regions 1 and 3 of the switch and the $\mathsf {A}$’s of the lock paired with the $i$ $\mathsf {U}$’s in region 4 of the switch. By expression 9, the total number of arcs is at most
  $$\begin{aligned}&{\text {MAC}}{}- 4k -i+ \max \{k,j\} + i= {\text {MAC}}{}- 4k + 2k\\&\quad = {\text {MAC}}{}- 2k. \end{aligned}$$
- $q= 2$, $c= 5$ and $j\le k$ The number of trans-arcs is at most $2k+ j$ (the total number of lock positions involved in trans-arcs). By expression 9, the total number of arcs is at most
  $$\begin{aligned}&{\text {MAC}}{}- 5k -i+ \max \{k,j\} + 2k = {\text {MAC}}{}- 5k -i\\&\quad + \, k + 2k \le {\text {MAC}}{}- 2k. \end{aligned}$$
- $q= 2$, $c= 5$ and $j> k$ The number of trans-arcs is at most $i+ j$; this bound is attained when the two regions of the lock that are involved in trans-arcs contain $\mathsf {C}$’s followed by $\mathsf {A}$’s, with the $j$ $\mathsf {A}$’s of the lock paired with $\mathsf {U}$’s in regions 2 and 4 of the switch and the $\mathsf {C}$’s of the lock paired with the $i$ $\mathsf {G}$’s in region 5 of the switch. By expression 9, the total number of arcs is at most
  $$\begin{aligned}&{\text {MAC}}{}- 5k -i+ \max \{k,j\} + i= {\text {MAC}}{}- 5k + j\\&\quad \le {\text {MAC}}{}- 2k. \end{aligned}$$
- $q= 2$ and $c\ge 6$ The number of trans-arcs is at most $2k+j$ where $j\le 2k$, and so the total number of arcs is at most
  $$\begin{aligned}&{\text {MAC}}{}- 6k -i+ \max \{k,j\} + 2k \le {\text {MAC}}{}- 6k + 2k\\&\quad +\, 2k = {\text {MAC}}{}- 2k. \end{aligned}$$
- $$q= 3 $$ and $$c\ge 2$$ Applying Claims 15 and 16, we have the following bound on the number of arcs
  $$\begin{aligned}&{\text {MAC}}{}_{\mathrm {switch}}(k,A){}+ t+ {\text {MAC}}{}_{\mathrm {lock}}(k,B){}- qk\\&\quad + \max \{k-j,0\} = {\text {MAC}}{}- 3k + \max \{k-j+ t,t\} \end{aligned}$$
  Since $t\le k$, if $j\ge t$ then the above expression can be bounded by ${\text {MAC}}{}- 2k$. Now, assume that $j < t\le k$. Then region 1 of the switch must form arcs with both regions $l_{b- 2}$ and $l_{b}$ of the lock. Since $l_{b- 2}$ forms arcs also with $L_{c}$, $c$ must be odd. Consider two subcases:
  - $c= 3$ The number of trans-arcs is at most $k + i$ (since $L_{2}$ can form any arcs). By expression 9, the total number of arcs is at most
    $$\begin{aligned}&{\text {MAC}}{}- 4k -i+ \max \{k-j,0\} + k + i\\&\quad \le {\text {MAC}}{}- 4k + k + k = {\text {MAC}}{}- 2k. \end{aligned}$$
  - $c\ge 5$ The number of trans-arcs is at most $2k + j$ (since $l_{b- 1}$ can form any arcs). By expression 9, the total number of arcs is at most
    $$\begin{aligned}&{\text {MAC}}{}- 6k -i+ \max \{k,j\} + 2k\\&\quad \le {\text {MAC}}{}- 6k + k + 2k < {\text {MAC}}{}- 2k. \end{aligned}$$
- $q\ge 4$: Applying Claim 15 while noting that $t\le k$, and applying also Claim 16, we have that the total number of arcs is at most
  $$\begin{aligned}&{\text {MAC}}{}_{\mathrm {switch}}(k,A){}+ k + {\text {MAC}}{}_{\mathrm {lock}}(k,B){}- qk \\&\qquad + \max \{k-j,0\} \le {\text {MAC}}{}+ k -4k + k\\&\quad = {\text {MAC}}{}-2k. \end{aligned}$$

$$\square $$

##### Corollary 7

*Let* $S$ *be a structure of* ${\text {switch-lock}}(k,A, B)$. *If* ${\text {AC}}(S) > {\text {MAC}}{}(k,A, B){}- 2k$, *then the number of trans-arcs in* $S$ *is at most* $k + t$.

##### Proof

By Claim 5, at most the first three left or the first three right regions of the switch are involved in trans-arcs. If this number less than three, the claim follows easily.

If this number is three, then by Claim 5, the number of regions in the lock involved in trans-arcs is one, hence, only the first and third regions of the switch can be involved in trans-arcs and the claim follows. $\square$

If not all intra-switch arcs are in the left or all in the right position, we can prove a slightly stronger version of Claim 15.

##### Claim 17

Consider a structure $S$ of ${\text {switch-lock}}(k,A, B)$ such that ${\text {AC}}(S) > {\text {MAC}}{}(k,A, B){}-2k$. If there are left–left trans-arcs and in addition, not all intra-switch arcs are in the right position, then the number of arcs involving bases of the switch is at most ${\text {MAC}}{}_{\mathrm {switch}}(k,A){}$.

Similarly, if there are right–right trans-arcs and in addition, not all intra-switch arcs are in the left position, then the number of arcs involving bases of the switch is at most ${\text {MAC}}{}_{\mathrm {switch}}(k,A){}$.

##### Proof

We consider the case where there are left–left trans-arcs. The right–right case is symmetric. In this case the bases of the right part of the switch can be involved only in the intra-switch arcs, hence, the number of arcs involving bases of the switch equals the number of paired bases of the left part of the switch. The claim follows if we show that there are at least $k$ bases in the left part of the switch that are unpaired. Let the outermost switch arc that is not in the right position go from region $L_\sigma$ to region $R_{\sigma '}$, where $\sigma \ne \sigma ' + 1$. We must have $\sigma \ge \sigma ' + 3$ or $\sigma \le \sigma ' - 1$.

First suppose that $$\sigma \ge \sigma ' + 3\ge 4$$. Consider arcs with one endpoint to the left of region $$L_{\sigma }$$. Consider two subcases. The first is when all such arcs are trans-arcs. By Corollary 7, there are at most $$2 k $$ trans-arcs. Hence, at least $$ k $$ bases in regions between $$L_{1}$$ and $$L_{\sigma - 1 }$$ are unpaired. The second subcase is when there is an intra-switch arc with an endpoint to the left of region $$L_{\sigma }$$. Consider the innermost such an arc. Suppose that this arc goes from region $$L_{\rho }$$ to region $$R_{\rho '}$$, where $$ \rho = \rho ' + 1$$ (since the arc is in the right position), and clearly $$\rho ' \le \sigma ' $$. Thus, $$ \rho = \rho ' + 1\le \sigma ' +1 \le \sigma -2$$. It follows that $$ k $$ bases of region $$L_{\sigma - 1}$$ are unpaired, because the structure cannot have pseudoknots.

Next suppose that $\sigma \le \sigma ' - 1$. Again we will show that there exists a region between $L_{\sigma +1}$ and $L_A$, having all bases are unpaired. Suppose that at least one base in each region from $L_{\sigma +1}$ to $L_{A-1}$ is paired. These must be paired to bases in regions $R_{\sigma ' +1}$ to $R_{A}$ respectively, in which case no base in region $L_{A}$ can paired and we are done. $\square$

### Bounding the arc count in structures with off-center arcs

#### Claim 18

Consider a structure $S$ of ${\text {switch-lock}}(k,A, B)$ with an on-center arc $\alpha$ from the $i$-th leftmost base of left region $\sigma$ of the lock to the $j$-th rightmost base of the right region $\sigma$. Then the number of arcs of $S$ that lie outside $\alpha$ (i.e., that $\alpha$ does not cover) is at most $X := {\text {MAC}}{}_{\mathrm {switch}}(k,A){}+ (\sigma - 1)2k + \max \{i,j\} - 1$.

#### Proof

Assume that there are more than $X$ such arcs. Consider structure $S'$ constructed from $S$ by replacing all arcs inside $\alpha$ with all possible on-center arcs. The number of these arcs is ${\text {MAC}}{}_{\mathrm {lock}}(k,B){}- 2k\sigma + \min \{2k - i,2k - j\} = {\text {MAC}}{}_{\mathrm {lock}}(k,B){}+(1 - \sigma )2k - \max \{i,j\}$. Hence, the total number of arcs of $S'$ is more than ${\text {MAC}}{}_{\mathrm {switch}}(k,A){}+ (\sigma - 1)2k + \max \{i,j\} - 1 + 1 + {\text {MAC}}{}_{\mathrm {lock}}(k,B){}+ (1 - \sigma )2k - \max \{i,j\} = {\text {MAC}}{}$, a contradiction with Claim 3. $\square$

#### Claim (6)

Let $S$ be a structure for ${\text {switch-lock}}(k,A, B)$, in which an on-center arc covers an off-center arc. Then ${\text {AC}}(S)\le {\text {MAC}}{}(k,A, B){}- 2k$.

#### Proof

Let $\alpha$ be an on-center arc from the $i$-th leftmost base of left region $\sigma$ of the lock to the $j$-th rightmost base of the right region $\sigma$ that covers an off-center arc. Let $\alpha '$ be the outermost such off-center arc, extending from left region $\tau$ to right region $\tau '$ of the lock. Without loss of generality, we can assume $\tau < \tau '$, i.e., $\tau \le \tau ' - 2$. Note that $\sigma \le \tau$. We count the number of arcs of $S$ as follows.

1. The number of arcs that lie between left region $\tau$ and right region $\tau '$ inclusive is at most ${\text {MAC}}{}_{\mathrm {lock}}(k,B){}+ (1 - \tau ')2k$. All other arcs that are covered by $\alpha$ are on-center arcs.
2. The number of on-center arcs that are covered by $\alpha$ and not covered by $\alpha '$ is at most $(\tau - \sigma )2k + \min \{2k - i,2k - j\} = (\tau - \sigma + 1)2k + \min \{-i,-j\}\le (\tau ' - \sigma - 1)2k - \max \{i,j\}$ (since $\tau \le \tau ' - 2$).
3. The number of arcs that are not covered by $\alpha$ (including $\alpha$ itself) is at most ${\text {MAC}}{}_{\mathrm {switch}}(k,A){}+ (\sigma - 1)2k + \max \{i,j\}$, by Claim 18.

Thus the total number of arcs of $S$ is at most ${\text {MAC}}{}(k,A, B){}+ 2k((1 - \tau ') + (\tau ' - \sigma - 1) + (\sigma - 1)) = {\text {MAC}}{}(k,A, B){}- 2k$.

#### Corollary (1)

Let $S$ be a structure for *switch and lock sequence* ${\text {switch-lock}}(k,A, B)$ *with* ${\text {AC}}(S) > {\text {MAC}}{}(k,A, B){} - 2k$, *such that* $S$ *has an on-center arc* $\alpha$ *between regions* $l_\sigma$ *and* $r_\sigma$ *of the lock. Then for every* $\sigma ' , \sigma < \sigma ' \le B$, *there is at least one on-center arc from lock region* $l_{\sigma '}$ *to lock region* $r_{\sigma ' }$.

#### Proof

Assume $\alpha$ connects the $i$-th leftmost base of region $l_\sigma$ with the $j$-th rightmost base of region $r_\sigma$. Assume that for some $\sigma ' \in \{\sigma + 1,\dots ,B\}$, there are no on-center arcs from $l_{\sigma ' }$ to $r_{\sigma ' }$. Since by the contrapositive of Claim 6, all arcs covered by $\alpha$ are on-center arcs, bases in regions $l_{\sigma ' }$ and $r_{\sigma ' }$ are not involved in any arcs. Hence, the number of arcs of $S$ covered by $\alpha$ is at most ${\text {MAC}}{}_{\mathrm {lock}}(k,B){}- 2k \sigma - \max \{i,j\}$. This contradicts Claim 18 and the fact that ${\text {AC}}(S) > {\text {MAC}}{}(k,A, B){}- 2k$. $\square$

### The main proof

#### Claim (8)

Suppose that $i$ is such that ${\text {AC}}(S_{p_{i}}) > {\text {MAC}}{}(k,A, B){}- 2k$. If $i$ is odd, then all intra-switch arcs must be in the right position and if $i$ is even, all intra-switch arcs must be in the left position.

#### Proof

Suppose that $i$ is odd (the case where $i$ is even is similar), not all intra-switch arcs are in the right position and that ${\text {AC}}(S_{p_{i} - 1}),{\text {AC}}(S_{p_{i}}) > {\text {MAC}}{}(k,A, B){}- 2k$. We obtain a contradiction by showing how to construct a structure $S$ with more than ${\text {MAC}}{}(k,A, B){}$ arcs. There are several cases:

- $$S_{p_i}$$ *has an off-center arc from* $$l_i$$ By Claim 6, this off-center arc must have its endpoint in a region $$r_j$$ where $$j\le i-2$$, since otherwise structure $$S_{p_i-1}$$ would have an off-center arc covered by an on-center arc. This also implies that in structure $$S_{p_i}$$, region $$r_i$$ is not involved in arcs: by definition of $$S_{p_i}$$ there are no on-center arcs to bases in $$r_i$$, and there cannot be off-center arcs either since these would be covered by an on-center arc in $$S_{p_i-1}$$, again contradicting Claim 6. Additionally, in structure $$S_{p_i}$$, region $$r_{i-1}$$ is not involved in arcs, since in $$S_{p_i-1}$$, any such arc would create a pseudoknot either with the off-center arc from $$l_i$$ or with the on-center arc from $$l_i$$. Then to obtain $$ S $$ we “shift” center arcs and trans-arcs of $$S_{p_i}$$ that have a right endpoint to the right of region $$r_{i-1}$$ of the lock. That is, we remove any arc $$u\cdot r_{a,j}$$ where $$a<i-1$$ and replace it by $$u\cdot r_{a+2,j}$$. We can then add $$2 k $$ $$\mathsf {P}\cdot \mathsf {Q}$$-arcs to obtain structure $$ S $$.
- $$S_{p_i}$$ *has an off-center arc from* $$r_i$$ In a manner that is symmetric to the previous case, we can shift center and trans-arcs of $$S_{p_i}$$ that have a left endpoint to the left of region $$l_{i-1}$$ of the lock and add $$2 k $$ $$\mathsf {X}\cdot \mathsf {Y}$$-arcs to obtain structure $$ S $$.
- $$S_{p_i}$$ has no off-center arcs and no trans-arcs to $$l_i$$ or $$r_i$$. We can simply add $$2 k $$ arcs from $$l_i$$ to $$r_i$$ to obtain $$ S $$.
- $$S_{p_i}$$ *has no off-center arcs to either* $$l_i$$ *or* $$r_i$$ *and has left–left trans-arcs to* $$l_i$$ Since not all intra-switch arcs are in the right position, by Claim  17, the total number of intra-switch plus trans-arcs is at most $${\text {MAC}}{}_{\mathrm {switch}}(k,A){}$$. To obtain $$ S $$ we can remove the trans-arcs from $$S_{p_i}$$, replace the switch structure with one that has $${\text {MAC}}{}_{\mathrm {switch}}(k,A){}$$ arcs, and add $$2 k $$ arcs from $$l_i$$ to $$r_i$$ to obtain $$ S $$.
- $S_{p_i}$ *has no off-center arcs to either* $l_i$ *or* $r_i$ *and has right–right trans-arcs to* $r_i$ Then the bases in the rightmost region of the switch (region $R_{1}$) cannot form intra-switch arcs, nor can they form a trans-arc with a base in some right lock region, say $r_j$, since then $j > i$ (recall $i$ is odd, hence, no base in $r_{i}$ can pair with a base in $R_{1}$), and this trans-arc would create a pseudoknot with the on-center arc from $l_i$ to $r_i$ in $S_{p_{i} - 1}$. Hence, all bases in the rightmost switch region are unpaired ($t = 0$) and the number of arcs involving bases of the switch is at most ${\text {MAC}}{}_{\mathrm {switch}}(k,A){}$, by Claim 15. To obtain $S$ we can remove the trans-arcs and proceed as in the previous case.$\square$

#### Corollary 8

*For every odd (even)* $i$, *if* ${\text {AC}}(S_{p_{i} - 1}),{\text {AC}}(S_{p_{i}}) > {\text {MAC}}{}(k,A, B){}- 2k$, *at least* $(A- 4)k$ *intra-switch arcs are in the right (left) position and no intra-switch arcs are in the left (right) position.*

#### Proof

Without loss of generality assume $i$ is odd. Recall that $t$ is the number of bases in the first left or first right switch regions involved in trans-arcs. By Corollary 7, the number of trans-arcs is at most $k + t$. Assume to the contrary that less than $(A- 4)k$ intra-switch arcs are in the right position. By Claim 8, there are less than $(A- 4)k$ intra-switch arcs. Hence, the total number of arcs involving bases of the switch is less than $(A- 4)k + k + t$. Consider a structure $S$ constructed from $S_{p_{i}}$ by removing all arcs involving bases in all switch regions other than the first left and the first right, and adding arcs under the following two conditions: (1) if $S_{p_{i}}$ contains right–right trans-arcs, add the arcs of the initial structure of the switch, or (2) otherwise, add the arcs of the target structure of the switch. It is easy to see that $S$ is a valid structure, hence, ${\text {AC}}(S)\le {\text {MAC}}{}(k,A, B){}$. Since $S$ was constructed by removing less than $(A- 4)k + k = {\text {MAC}}{}_{\mathrm {switch}}(k,A){}- 2k$ arcs and adding ${\text {MAC}}{}_{\mathrm {switch}}(k,A){}$ arcs, we have ${\text {AC}}(S) > {\text {AC}}(S_{p_{i}}) - ({\text {MAC}}{}_{\mathrm {switch}}(k,A){}- 2k) + {\text {MAC}}{}_{\mathrm {switch}}(k,A){}= {\text {AC}}(S_{p_{i}}) + 2k$. It follows by Claim 3 that ${\text {AC}}(S_{p_{i}}) < {\text {MAC}}{}(k,A, B){}- 2k$, which is a contradiction. $\square$

#### Corollary (2)

*If* ${\text {AC}}(S_{p_{i} - 1}),{\text {AC}}(S_{p_{i}}) ,{\text {AC}}(S_{p_{i + 1} - 1}),{\text {AC}} (S_{p_{i + 1}})> {\text {MAC}}{}(k,A, B){}- 2k$, *the number of steps (i.e., structures in the pathway* $P$) *from* $S_{p_i}$ *to* $S_{p_{i+1}}$ *is at least* $2(A- 4)k$.

#### Proof

By Corollary 8, at least $(A- 4)k$ arcs needs to be removed and at least $(A- 4)k$ arcs needs to be added. Hence, the number of steps is at least $2(A- 4)k$. $\square$

## The 4-letter alphabet design using the stacked base pair energy model

### General results

#### Claim (9)

Let $s$ be a sequence over the 8-letter alphabet. Let $S'$ be a structure for $s' = {\text {map}}(s)$. For any eccentric stacked arc pair of $S'$ between positions $i$ and $j$, and $i + 1$ and $j - 1$, either pair $i,i + 1$ or pair $j - 1,j$ is a boundary.

#### Proof

Consider an eccentric stacked arc pair between positions $i$ and $j$, and $i + 1$ and $j - 1$. Assume that neither $i,i + 1$ or $j - 1,j$ is a boundary, i.e., positions $i$ and $i + 1$ (respectively, $j - 1$ and $j$) lie in the same region. It is easy to check that these two regions must be complementary, as two consecutive bases of any region identify the type of region. For instance, $\mathsf {AC}$ or $\mathsf {CA}$ lie only inside an $\mathsf {X}$-region. Hence, these two stacked arcs are not eccentric, a proof by contrapositive. $\square$

#### Theorem (2)

*Let* $$ s $$ *be a sequence over the 8-letter alphabet and let* $$s' = {\text {map}}(s)$$. *Assume that any structure for* $$s'$$ *with at least* $${\text {MAC}}{}(s) - (K - E)$$ *stacked arc pairs has at most* $$ E $$ *eccentric stacked arc pairs. Let* $$S_1'$$ *and* $$S_2'$$ *be two structures of* $$s'$$, *and let* $$S_1 = {\mathrm{Map}^{\prime }}(S_1')$$ *and* $$S_2 = {\mathrm{Map}^{\prime }}(S_2')$$ *be structures of* $$ s $$. *Let* $$D = {\text {SAC}}(S_{1}') - {\text {MAC}}{}(s)$$. *Suppose that any pathway between* $$S_{1}$$ *and* $$S_{2}$$ *with barrier at most* $$ K $$ *has length at least* $$ L $$. *Then any pathway between* $$S_1'$$ *and* $$S_2'$$ *with barrier at most* $$K + D - E$$ *has length at least* $$ L /2$$.

#### Proof

Assume to the contrary that there is a pathway $$P'$$ from $$S_{1}'$$ to $$S_{2}'$$ with barrier at most $$K + D - E$$ of length less than $$ L /2$$. We will construct a pathway $$ P $$ from $$S_{1}$$ to $$S_{2}$$ of length at most twice the length of $$P'$$ with barrier at most $$ K $$. We will do so in two steps. First, map each structure $$S'$$ in $$P'$$ to the structure for $$ s $$ using the mapping $${\mathrm{Map}^{\prime }}()$$, thus obtaining a sequence of structures $$ P $$. Second, we transform $$ P $$ to a proper pathway between $${\mathrm{Map}^{\prime }}(S_{1}')$$ and $${\mathrm{Map}^{\prime }}(S_{2}')$$, so that any consecutive structures in $$ P $$ differ by exactly one arc. Consider two consecutive structures of the sequence $$ P $$. They differ by at most two arcs. This is because, each added (removed) arc in $$P'$$ can create (remove) at most two stacked arc pairs. Consequently, we need to insert at most one intermediate structure between these two consecutive structures to make $$ P $$ a proper pathway, thus increasing the length of $$ P $$ by at most $$|P'|-1$$, i.e., $$|P| < 2|P'|$$. Some consecutive structures of $$ P $$ might be identical (for instance, if an eccentric arc was added in $$P'$$). In this case we omit one of the repeated structures in $$ P $$.

Since $P'$ has barrier at most $K + D - E$, for any structure $S'$ of $P'$, ${\text {SAC}}(S_{1}') - {\text {SAC}}(S')\le K + D - E$. Hence, ${\text {SAC}}(S')\ge {\text {SAC}}(S'_{1}) - (K + D - E) = {\text {MAC}}{}(s) - (K - E)$. Thus, by the assumption in the theorem statement, $S'$ has at most $E$ eccentric stacked arc pairs. Hence by Claim 10, we have for the corresponding structure $S = {\mathrm{Map}^{\prime }}(S')$ that ${\text {MAC}}{}(s) - {\text {AC}}(S)\le {\text {MAC}}{}(s) - ({\text {SAC}}(S') - E)\le K$. Clearly, this bound also applies to the structures which were inserted to $P$. Hence, $P$ has a barrier at most $K$ and length at most $2|P'| -1 < L$, a contradiction. $\square$

#### Corollary (5)

*Let* $B$ *be even. Let* $s' = {\text {map}}({\text {switch-lock}}(k,A, B))$. *Then there exists a pathway from the initial to the target structure of* $s'$ *with barrier* $k + 3$ *and with length* $(2(k + 1)(A+ 2) - 3)B+2(k + 1)(A- 1)$. *The length of any pathway from the initial to the target structure with barrier at most* $2k - A- 6B- 2$ *is at least* $k(A- 4)(B- 1)$.

#### Proof

To prove the first part of the corollary we construct a pathway between the initial structure $S_{1}'$ and target structure $S_{2}'$ by reusing the pathway for the 8-letter alphabet described in the proof of Theorem 1. However, we have one more base in each region. Consequently, switching the switch will require $2(k + 1)(A- 1)$ steps. While unlocking the lock, in phases (iii), (iv) and (vi) one additional arc will be removed/added, hence, the number of steps to unlock each band of the lock is $2(k + 1)(A- 1) + 6k + 3$. The total length of the pathway is $(2(k + 1)(A+ 2) - 3)B+2(k + 1)(A- 1)$. For any structure $S'$ on this pathway, we have ${\text {SAC}}(S_{1}')\ge {\text {SAC}}(S')\ge {\text {SAC}}(S_{1}') - (k + 3)$, since in $S'$ either arcs of the switch are split two to stacked blocks of arcs (one block in the left position and the other block in the right position), in which case, the count of stacked arc pairs is decreased by $k + 3$, or the switch is in the initial or target position and lock is unlocked in phases (i)-(vi), in which the count of stacked arc pairs is decreased by at most $k$. Hence, the barrier of this pathway is $k + 3$.

Let $S_{1}'$ and $S_{2}'$ be the initial and target structures for $s'$ respectively. Note that ${\mathrm{Map}^{\prime }}(S_{1}')$ and ${\mathrm{Map}^{\prime }}(S_{2}')$ are the initial and target structures for ${\text {switch-lock}}(k,A, B)$. Since $s'$ has $2A+ 6 B$ regions, by Corollary 4, any structure for $s'$ has at most $2A+ 6 B- 1$ eccentric stacked arc pairs. Structure $S_{1}'$ has exactly $A- 2$ eccentric stacked arc pairs. Hence, by Claim 10, $D = {\text {SAC}}(S_{1}') - {\text {MAC}}{}(s) = {\text {AC}}(S_{1}) + A- 2 - {\text {MAC}}{}(s) = A- 2$, since $S_{1}$ is an MFE structure for $s$. The second part of the claim thus follows by Theorems 1 and 2 by setting $K = 2k - 1$, $E = 2A+ 6 B- 1$, $D = A- 2$ and $L = 2k(A- 4)(B- 1)$. $\square$

### The switch and lock sequence

We will need the following terminology. A stacked arc pair that connects a boundary between regions $r$ and $r'$ to a region $r''$ will be called an “$(r,r')\cdot r''$-stacked arc pair”.

#### Claim (11)

Consider a structure $S'$ for ${\text {lock}}^{\prime }(k, B)$. Then the number of non-eccentric stacked arc pairs in $S'$ is at most $(2k + 1)B$.

#### Proof

Since we are only counting non-eccentric stacked arc pairs, we only need to consider non-eccentric arcs. To count the number of non-eccentric arcs, we can use the argument in the proof of Claim 2. Considering the different sizes of regions ($k + 1$ for $p , q , x , y$-regions and $2k + 1$ for $l , r$-regions), Bound (8) on the number of non-eccentric arcs becomes

$$\begin{aligned}&(k + 1)i + (k + 1)j + (2k + 1)(B- \max \{i,j\})\\&\quad \le (2k + 1)B+ (i + j)/2 \le (2k + 2)B, \end{aligned}$$

where the last inequality follows since $i,j\le B$. Since the longest possible stacked run of arcs in the lock has length $2k + 1$, the maximum number of non-eccentric stacked arc pairs these arcs can create is at most $\frac{2k}{2k + 1}(2k + 2)B< (2k + 1)B$. $\square$

#### Claim (12)

For any structure $S'$ of ${\text {switch-lock}}^{\prime }(k,A, B)$ with at least ${\text {MAC}}{}(k,A, B){}- 2k + 8B+ 4$ stacked arc pairs, the number of its eccentric stacked arc pairs is at most $8B+ 2$.

#### Proof

Let $S'$ be a structure for ${\text {switch-lock}}^{\prime }(k,A, B)$ with at least ${\text {MAC}}{}(k,A, B){}- 2k + 8B+ 4$ stacked arc pairs. Without loss of generality we can assume that $S'$ does not contain any arcs that are not stacked. First note that there are no intra-switch eccentric stacked arc pairs in $S'$. Second, the number of eccentric stacked arc pairs in $S'$ that involve a boundary that contains at least one base of the lock is at most $6B$. It remains to show that the number of eccentric stacked arc pairs that do not involve a boundary in the lock is at most $2B+ 2$. Assume to the contrary that the number of such eccentric stacked arc pairs is at least $2B+ 3$. Note that all these eccentric stacked arc pairs must involve a boundary in the switch. There are only three types of such stacked arc pairs since they are only three types of boundaries in the switch and each of them can form a base pair with only one type of regions in the lock (recall that two consecutive bases determine the type of a region):

1. $(L_{i},L_{i + 1})\cdot x_{j}$-stacked arc pairs,
2. $(L_{A}, R_{A})\cdot p_{j}$-stacked arc pair, and
3. $(R_{i},R_{i - 1})\cdot y_{j}$-stacked arc pairs.

The stacked arc pairs of type 1. cross stacked arc pairs of type 2. and 3. and there is at most one stacked arc pair of type 2.

Consider the case when $S'$ contains stacked arc pairs of type 1 (and hence, none of type 2 and 3). Let $d$ be the maximal index such that $(L_{d},L_{d + 1})\cdot x_{j}$ is in $S'$ for some index $j$. We must have $d\ge 2B+ 3$. Note that the bases of $L_{1},\dots ,L_{d}$ and the first base of $L_{d + 1}$ can only form trans-arcs and the remaining bases of the switch can only form intra-switch arcs or eccentric stacked arc pairs of type $(L_{i}\cdot (x_{j'},x_{j' + 1}))$ which are already counted (in $6B$). Consider trans-arcs involving bases of $L_{1},\dots ,L_{d}$ and the first base of $L_{d + 1}$ that do not involve boundaries between $x$-regions, i.e., are not parts of stacked arc pairs of type 1. These trans-arcs must involve bases of $l$-regions of the lock, hence, their number is at most $(2k + 1)B$. Since the longest possible stacked run of such trans-arcs has length at most $k$, the number of stacked arc pairs that do not involve boundaries in the lock is at most $\frac{k - 1}{k}(2k + 1)B\le 2kB$.

The number of intra-switch stacked arc pairs is at most $k(A- d) - 2$. Hence, the total number of stacked arc pairs involving bases of the switch and not involving boundaries in the lock is at most

$$\begin{aligned} 2kB+ k(A- d) - 2 \le 2kB+ k(A- 2B- 3) - 2 = k(A- 3) - 2. \end{aligned}$$

Since any stacked arc pair in $S'$ involves a boundary in the lock (at most $6B$ of them), or involves bases of the switch and does not involve a boundary in the lock (at most $k(A- 3) - 2$ of them), or is intra-lock and does not involve a boundary in the lock, i.e., is non-eccentric intra-lock (at most $(2k + 1)B$ of them by Claim 11). It follows that

$$\begin{aligned} {\text {SAC}}(S')&\le 6B+ k(A- 3) - 2 + (2k + 1)B\\&= {\text {MAC}}{}(k,A, B){}- 2k + 7B- 2\\&\le {\text {MAC}}{}(k,A, B){}- 2k + 8B+ 3\,, \end{aligned}$$

a contradiction. Hence, the number of eccentric stacked arc pairs is at most $8B+ 2$.

The proof in the case when $S'$ contains stacked arc pairs of type 2. and 3. is analogous. $\square$

#### Theorem (3)

Consider the sequence ${\text {switch-lock}}^{\prime }(k,A, B)$. There is a pathway from the initial to the target structure with barrier $k + 2$ and with length $(2k(A- 1) + 6k + 3)B+2k(A- 1 )$. Moreover, any pathway from the initial to the target structure of ${\text {switch-lock}}^{\prime }(k,A, B)$ with barrier at most $2k - 8B- 5$ has length at least $k(A- 4)(B- 1) - 1$.

#### Proof

The first part of the claim can be proved in a similar way as the first part of Corollary 5 (the barrier decreases by one, since the regions in the switch are shorter by one arc).

Consider a pathway $P$ from ${\mathrm{Map}^{\prime }}(S_{1}')$ to ${\mathrm{Map}^{\prime }}(S_{2}')$ with barrier at most $2k - 2$. Note ${\mathrm{Map}^{\prime }}(S_{1}')$ (${\mathrm{Map}^{\prime }}(S_{2}')$) is the initial (target) structure for $s = {\text {switch-lock}}(k,A, B)$ without the innermost arc. Appending the initial (target) structure for $S$ to the beginning (end) of $P$ we obtain a pathway from the initial to the target structure of $s$ with barrier at most $2k - 1$. By Theorem 1, this pathway has length at least $2k(A- 4)(B- 1)$. Hence, $P$ has length at least $2k(A- 4)(B- 1) - 2$.

Since $S_{1}'$ has no eccentric stacked arc pairs, ${\text {SAC}}(S_{1}') = {\text {AC}}({\mathrm{Map}^{\prime }}(S_{1}'))$, and hence, $D = {\text {SAC}}(S_{1}') - {\text {MAC}}{}(s) = {\text {AC}}({\mathrm{Map}^{\prime }}(S_{1}')) - {\text {MAC}}{}(s) = -1$. Now, the second part of the claim follows by Theorem 2 and Claim 12 by setting $D = -1$, $K = 2k - 2$, $E = 8B+ 2$ and $L = 2k(A- 4)(B- 1) - 2$. $\square$

## References

[CR1] Andronescu M, Fejes AP, Hutter F, Condon A, Hoos HH (2004). A new algorithm for RNA secondary structure design. J Mol Biol.

[CR2] Babitzke P, Yanofsky C (1993). Reconstitution of Bacillus subtilis Trp attenuation in vitro with TRAP, the Trp RNA-binding attenuation protein. Proc Natl Acad Sci USA.

[CR3] Beisel CL, Smolke CD (2009). Design principles for riboswitch function. PLoS Comput Biol.

[CR4] Busch A, Backofen R (2006). INFO-RNA–a fast approach to inverse RNA folding. Bioinform Adv Access.

[CR5] Condon A, Hu AJ, Maňuch J, Thachuk C (2016). Less haste, less waste: on recycling and its limits in strand displacement systems. J R Soc Interface Focus.

[CR6] Dirks RM, Lin M, Winfree E, Pierce NA (2004). Paradigms for computational nucleic acid design. Nucleic Acid Res.

[CR7] Dotu I, Lorenz WA, Van Hentenryck P, Clote P (2010). Computing folding pathways between RNA secondary structures. Nucleic Acids Res.

[CR8] Flamm C, Fontana W, Hofacker IL, Schuster P (2000). RNA folding at elementary step resolution. RNA.

[CR9] Geary C, Rothemund PWK, Andersen ES (2014). A single-stranded architecture for cotranscriptional folding of RNA nanostructures. Science.

[CR10] Geary CW, Andersen ES (2014) Design principles for single-stranded RNA origami structures. In: 20th International conference on DNA computing and molecular programming (Lecture notes in computer science), vol 8727. Springer, pp 1–19

[CR11] Gultyaev AP, Batenburg FH, Pleij CW (1998). Dynamic competition between alternative structures in viroid RNAs simulated by an RNA folding algorithm. J Mol Biol.

[CR12] Hagiya M, Yaegashi S, Takahashi K (2006) Computing with hairpins and secondary structures of DNA. In: Nanotechnology: science and computation. Springer, Berlin, pp 293–308

[CR13] Haleš J, Maňuch J, Ponty Y, Stacho L (2015) Combinatorial RNA design: designability and structure-approximating algorithm. In: Proceedings of combinatorial pattern matching (CPM, 2015), volume 9133 of LNCS, pp 231–246

[CR14] Isaacs FJ, Dwyer DJ, Collins JJ (2006). RNA synthetic biology. Nat Biotechnol.

[CR15] Jaeger L, Westhof E, Leontis NB (2001). TectoRNA: modular assembly units for the construction of RNA nano-objects. Nucleic Acid Res.

[CR16] Kuhlman B, O’Neill JW, Kim DE, Zhang KYJ, Baker D (2002). Accurate computer-based design of a new backbone conformation in the second turn of protein l. J Mol Biol.

[CR17] Lakin MR, Youssef S, Polo F, Emmott S, Phillips A (2011). Visual DSD: a design and analysis tool for DNA strand displacement systems. Bioinformatics.

[CR18] Leea J, Kladwangb W, Leea M, Cantub D, Azizyana M, Kimc H, Limpaechera A, Yoonc S, Treuillea A, Das R, Participants EteRNA (2014). RNA design rules from a massive open laboratory. Proc Nat Acad Sci USA.

[CR19] Mathieson L-A, Condon A (2015) On low energy barrier folding pathways for nucleic acid sequences. In: 21st International conference on DNA computing and molecular programming (Lecture notes in computer science), vol 9211. Springer, pp 181–193

[CR20] Morgan SR, Higgs PG (1998). Barrier heights between ground states in a model of RNA secondary structure. J Phys A Math General.

[CR21] Nauli S, Kuhlman B, Baker D (2001). Computer-based redesign of a protein folding pathway. Nat Struct Biol.

[CR22] Norris NR (1997). Markov chains, Cambridge series on statistical and probabilistic mathematics.

[CR23] Qian L, Soloveichik D, Winfree E (2011) Efficient turing-universal computation with DNA polymers. In: 17th International conference on computing and molecular programming (Lecture notes in computer science), vol 6937. Springer, pp 123–140

[CR24] Qian L, Winfree E (2011). Scaling up digital circuit computation with DNA strand displacement cascades. Science.

[CR25] Qian L, Winfree E, Bruck J (2011). Neural network computation with DNA strand displacement cascades. Nature.

[CR26] Schaeffer JM, Thachuk C, Winfree E (2015) Stochastic simulation of the kinetics of multiple interacting nucleic acid strands. In: 21st International conference on DNA computing and molecular programming (Lecture notes in computer science), vol 9211. Springer, pp 194–211

[CR27] Schultes EA, Bartel DP (2000). One sequence, two ribozymes: implications for the emergence of new ribozyme folds. Science.

[CR28] Schuster P, Fontana W, Stadler P, Hofacker IL (1994). From sequences to shapes and back: a case study in RNA secondary structures. Proc R Soc Lond.

[CR29] Seelig G, Soloveichik D, Zhang DY, Winfree E (2006). Enzyme-free nucleic acid logic circuits. Science.

[CR30] Simmel FC, Dittmer WU (2005). DNA nanodevices. Small.

[CR31] Soukup GA, Breaker RR (1999). Engeneering precision RNA molecular switches. Proc Natl Acad Sci USA.

[CR32] Thachuk C, Condon A (2012) Space and energy efficient computation with DNA strand displacement systems. In: 18th International conference on DNA computing and molecular programming (Lecture notes in computer science), vol 7433. Springer, pp 135–150

[CR33] Uejima H, Hagiya M (2004) Secondary structure design of multi-state DNA machines based on sequential structure transitions (Lecture notes in computer science), vol 2943. Springer, Berlin, pp 74–85

[CR34] Yin P, Choi HMT, Calvert CR, Pierce NA (2008). Programming biomolecular self-assembly pathways. Nature.

[CR35] Yurke B, Turberfield AJ, Mills AJ, Simmel FC, Neumann JL (2000). A DNA-fuelled molecular machine made of DNA. Nature.

[CR36] Zhou Y, Ponty Y, Vialette S, Waldispuhl J, Zhang Y, Denise A (2013) Flexible RNA design under structure and sequence constraints using formal languages. In: Proceedings of the international Conference on bioinformatics, computational biology and biomedical informatics, BCB’13, ACM, New York, pp 229–238

